# Discovery of GPR183 Agonists Based on an Antagonist Scaffold

**DOI:** 10.1002/cmdc.202100301

**Published:** 2021-08-03

**Authors:** Viktoria M. S. Kjær, Loukas Ieremias, Viktorija Daugvilaite, Michael Lückmann, Thomas M. Frimurer, Trond Ulven, Mette M. Rosenkilde, Jon Våbenø

**Affiliations:** ^1^ Department of Biomedical Sciences Faculty of Health and Medical Sciences University of Copenhagen Blegdamsvej 3B 2200 Copenhagen Denmark; ^2^ Department of Drug Design and Pharmacology Faculty of Health and Medical Sciences University of Copenhagen Jagtvej 162 2100 Copenhagen Denmark; ^3^ Novo Nordisk Foundation Center for Basic Metabolic Research University of Copenhagen Maersk Tower Blegdamsvej 3B 2200 Copenhagen Denmark; ^4^ Helgeland Hospital Trust Prestmarkveien 1 8800 Sandnessjøen Norway

**Keywords:** agonists, antagonists, drug discovery, GPR183, receptors

## Abstract

The G protein‐coupled receptor GPR183/EBI2, which is activated by oxysterols, is a therapeutic target for inflammatory and metabolic diseases where both antagonists and agonists are of potential interest. Using the piperazine diamide core of the known GPR183 antagonist (*E*)‐3‐(4‐bromophenyl)‐1‐(4‐(4‐methoxybenzoyl)piperazin‐1‐yl)prop‐2‐en‐1‐one (NIBR189) as starting point, we identified and sourced 79 structurally related compounds that were commercially available. *In vitro* screening of this compound collection using a Ca^2+^ mobilization assay resulted in the identification of 10 compounds with agonist properties. To enable establishment of initial structure‐activity relationship trends, these were supplemented with five in‐house compounds, two of which were also shown to be GPR183 agonists. Taken together, our findings suggest that the agonist activity of this compound series is dictated by the substitution pattern of one of the two distal phenyl rings, which functions as a molecular efficacy‐switch.

## Graphical abstract

**Change your stripes**: Based on screening of a focused library containing 79 compounds with structural resemblance to the known GPR183 *antagonist* NIBR189 (**5**), herein we report the discovery of a novel series of small‐molecule GPR183 *agonists*, thereby providing clues to a molecular efficacy‐switch for this compound class.

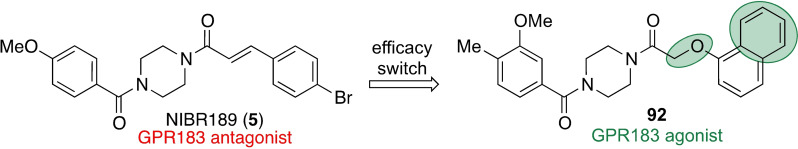

The G protein‐coupled receptor GPR183, also known as Epstein‐Barr virus (EBV) induced gene 2 (EBI2), was identified in 1993 as one of the most up‐regulated genes in EBV‐infected cells.^[1]^ Before its deorphanization, the constitutive activity of GPR183 facilitated the discovery of Gα_i_ coupling and ERK activation as downstream signaling events.^[2]^ These two pathways along with β‐arrestin coupling were subsequently shown to also be ligand‐induced.^[3]^ In 2011, GPR183 was deorphanized as two simultaneous papers revealed hydroxycholesterols (oxysterols) to be agonists of GPR183 with 7α,25‐dihydroxycholesterol (7α,25‐OHC, **1**) (Figure 1A) as the most potent endogenous ligand.^[4]^ The same two groups identified GPR183 as a chemotactic receptor with **1** acting as a potent chemoattractant, a finding that correlates well with the high expression of GPR183 in leukocytes^[2a]^ and the observation that GPR183 differential expression is important for correct B cell positioning within lymphoid organs.^[5]^

**Figure 1:**
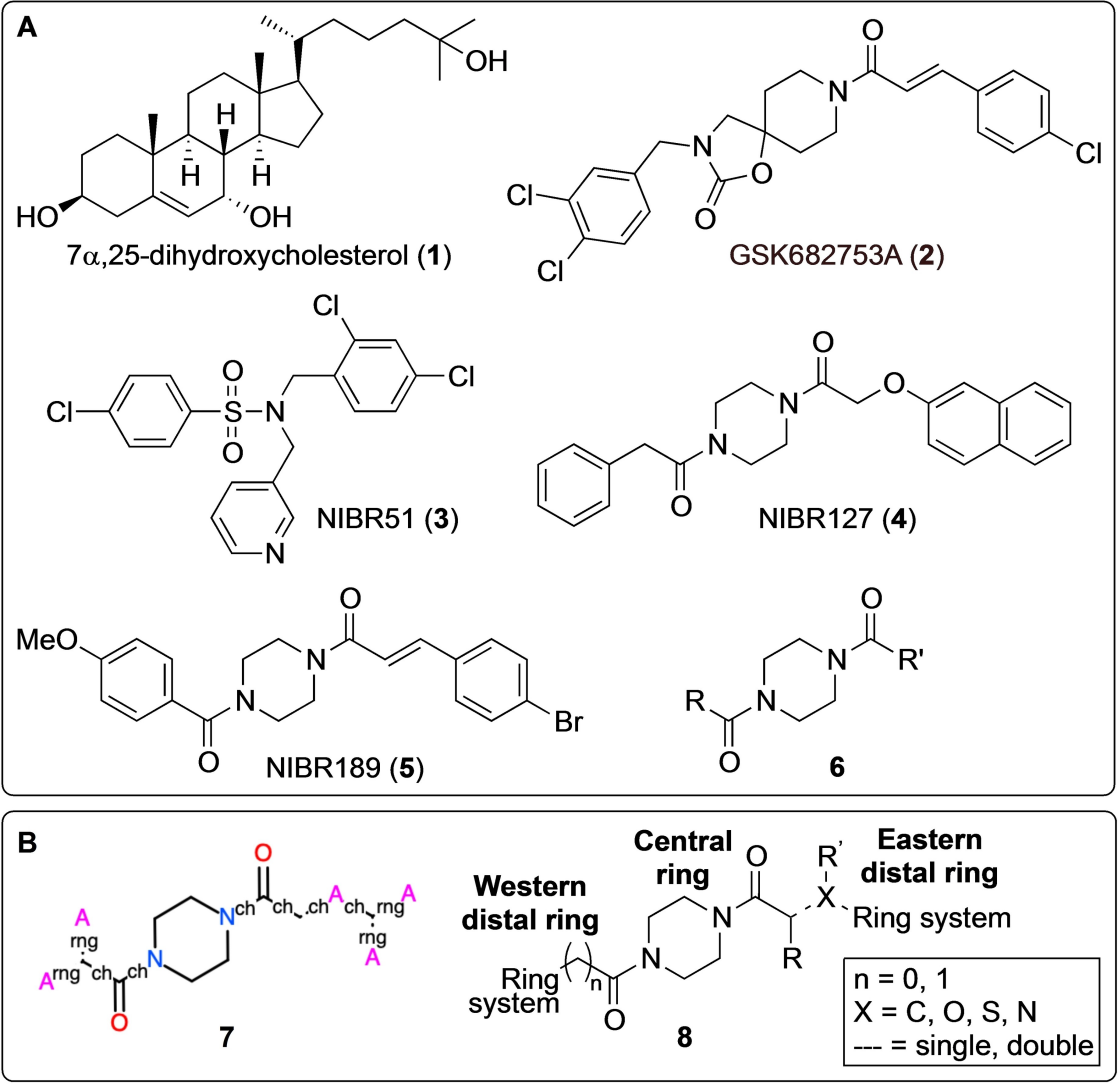
Known GPR183 ligands and general ligand structures. A) the endogenous agonist **1**, the reported synthetic ligands **2**–**5**, and the antagonist scaffold **6**, B) the Markush formula **7** used as search query (ch: chain bond, rng: ring bond, A: any atom) and the general structure **8** for the 79 screened compounds.

The pharmacological interest in GPR183 stems from the implication of the receptor and its endogenous ligands in a variety of diseases like B cell malignancies, inflammatory/autoimmune diseases and metabolic diseases.^[6]^ Moreover, it was recently shown to regulate interferons and bacterial growth during *Mycobacterium tuberculosis* infection.^[7]^

Efforts to identify synthetic GPR183 ligands have resulted in a handful of small molecules, including agonists as well as antagonists^[6a]^ (Figure 1A). Prior to the deorphanization of GPR183, a small molecule inverse agonist of GPR183 named GSK682753A (**2**) was identified; this compound was later shown to also inhibit 7α,25‐OHC (**1**)‐mediated GPR183 activity.[^2b^, ^3^] In 2014, screening of a library containing around 100 K compounds resulted in the discovery of the GPR183 agonist NIBR51 (**3**).^[8]^ This agonist was then used to rescreen the same compound library for GPR183 antagonists, leading to the identification of NIBR127 (**4**), which in turn was chemically optimized to give the more potent GPR183 antagonist NIBR189 (**5**).^[8]^ Using the molecular scaffold **6** (Figure 1A) of the known GPR183 antagonist **5** as starting point, we here report the discovery of a novel series of small molecule GPR183 agonists.

To identify commercially available compounds that were structurally related to the known GPR183 antagonist **5** ^[8]^ we first conducted a substructure screen of the Enamine Screening Collection (∼2.7 M compounds) using the Markush formula **7** (Figure 1B) as search query. The results were further filtered for “drug‐likeness” based on chemical criteria and subsequently clustered to ensure chemical diversity. A total of 79 compounds (**9**–**87**, supplementary Figure S1 and Table S1) were manually selected for experimental screening; these were all piperazine diamides of the general structure **8** (Figure 1B).

G protein activation induced by the 79 compounds was detected using CHO‐K1 cells that were transiently transfected with GPR183 and a chimeric Gα subunit Gqi4myr that is recognized as Gα_i_ by Gα_i_‐coupled receptors, but activates Gα_q_ pathways,^[9]^ consequently enabling Ca^2+^ release. Calcium release was measured for 100 seconds after ligand addition by utilizing a fluorescent indicator, and data was extracted as the change in fluorescence over time. Based on the G protein signaling efficacy induced by 10 μM of each compound (Figure 2, top), we selected 10 of the compounds (**9**–**18**) for further dose‐response experiments (Figure 2, middle). Here, compounds **15** and **16** displayed the most favorable agonist properties, combining acceptable efficacy and potency (EC_50_ of 209 nM and 179 nM, respectively). As previously shown, the antagonist **5** did not display any intrinsic activity, while the endogenous agonist **1** activated the receptor with a potency (EC_50_) of 17 nM (Figure 2, shown as reference curves).

**Figure 2:**
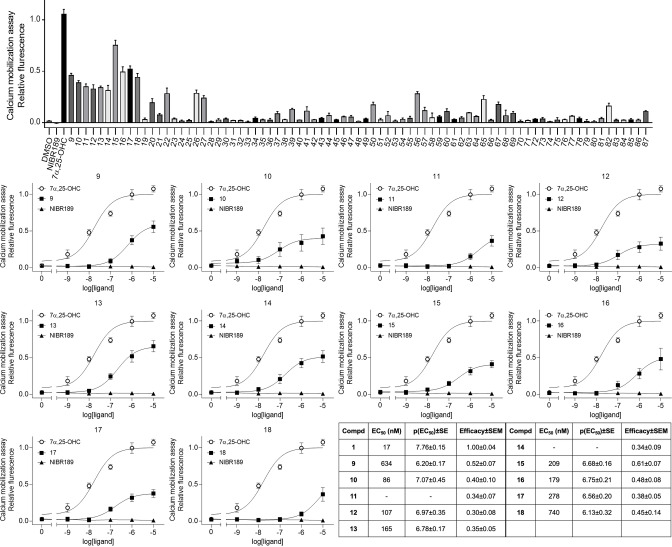
Top: Screening of compounds **9**–**87** in a calcium mobilization assay, with solvent (DMSO), the endogenous agonist **1**, and the known antagonist **5** included as controls. Calcium response determined upon addition of 10 μM compound and represented as relative fluorescence with 1 being the maximum response induced by **1**. Middle: Calcium assay dose‐response graphs of the 10 most active compounds (**9**–**18**) from the screen. Represented as relative fluorescence for each concentration used. Curves for compounds **1** and **5** added for reference. Bottom: Table showing potency (EC_50_), p(EC_50_)±SE and efficacy±SEM (determined as activity induced by 10 μM ligand) for compounds **9**–**18**. All data represent mean ±SEM of 3 individual experiments performed in duplicates.

As all the screened compounds were built on a central piperazine diamide core, the structural variation was in the two distal ring systems and the spacers (Figure 1B). Of the 10 active compounds (Figure 3), four contained the same (*E*)‐alkene spacer as the antagonist **5**, two contained an ethylene spacer, and four contained the oxy‐methylene spacer found in the known antagonist **4**. However, the non‐systematic structural variation in the distal ring systems made it difficult to identify clear structure‐activity relationship (SAR) trends in this compound series.

**Figure 3:**
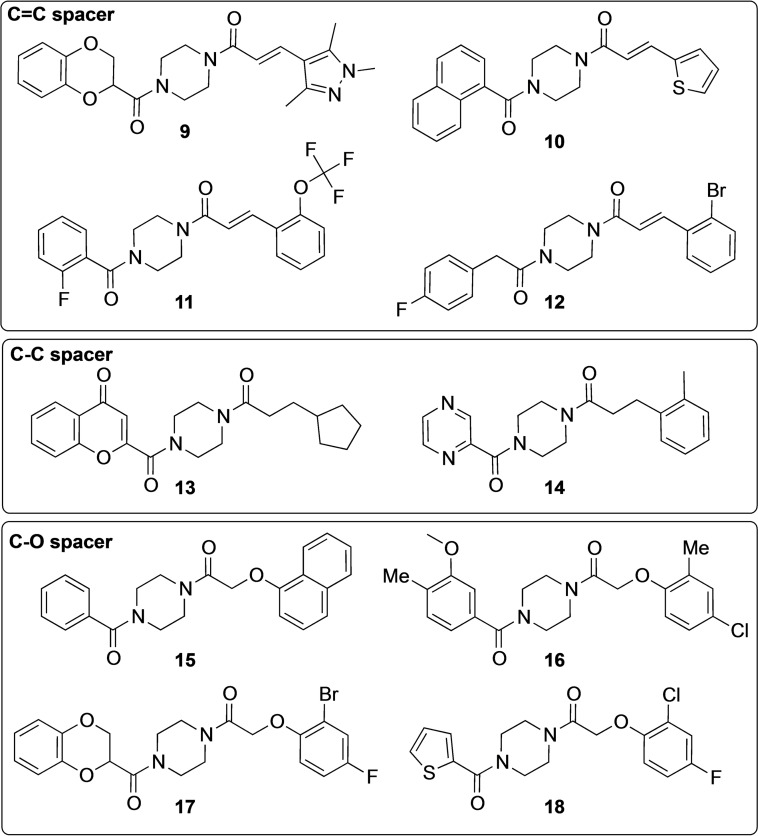
Structures of the 10 compounds (**9**–**18**) that showed agonistic activity in the initial screening, grouped by type of two‐atom spacer (C=C, C−C, C−O).

We therefore designed and synthesized five additional compounds (Scheme 1): three reference compounds (**88**–**90**) that contained distal unsubstituted phenyl rings, as well as two crossover compounds (**91** and **92**) that combined the Western and Eastern ring systems of the top agonist hits **15** and **16**.

**Scheme 1:**
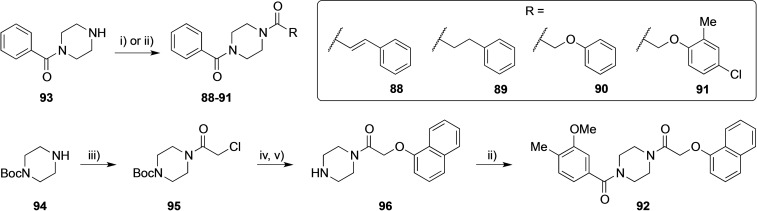
Synthesis of target compounds **88**–**92**. Reagents and conditions: i) PhCH=CHCOCl or Ph(CH_2_)_2_COCl, TEA, DCM, rt, 24 h, 84–90 %; ii) 2‐phenoxyacetic acid or 2‐(4‐chloro‐2‐methylphenoxy)acetic acid or 3‐methoxy‐4‐methylbenzoic acid, EDC HCl, DIPEA, DMF, 0 °C to rt, 24 h, 92–97 %; iii) 2‐chloroacetyl chloride, TEA, DCM, rt, 24 h, 99 %; iv) naphthalen‐1‐ol, Cs_2_CO_3_, KI, acetone, 35 °C, 24 h, 82 %; v) TFA:DCM, 1 : 1, rt, 1 h, 74 %.

The substituted piperazines **88** and **89** were synthesized in one step from commercially available phenyl(piperazin‐1‐yl)methanone (**93**) via coupling with the corresponding acyl chlorides (Scheme 1). The same starting material (**93**) was reacted with the corresponding carboxylic acids in the presence of EDC to afford **90** and **91** in excellent yields. Coupling of Boc‐piperazine (**94**) and chloroacetyl chloride yielded intermediate **95**. This was further reacted with 1‐naphthol and Boc‐deprotected to give **96**. Subsequent amide coupling employing 1‐ethyl‐3‐(3‐dimethylaminopropyl)carbodiimide (EDC) led to compound **92** in excellent yield.

As Gα_i_ is the direct G protein‐signaling pathway elicited by GPR183,^[2a]^ we switched to this pathway for the functional tests of the synthesized compounds **88**–**92**, and included **15** and **16** for comparison with the calcium release experiment in the initial screen. Hence, the GPR183 agonist activity of the five in‐house compounds was experimentally tested using a BRET assay to determine Gα_i_ coupling at various concentrations of the compounds. CHO‐K1 cells were transiently transfected with GPR183 and the CAMYEL (cAMP sensor using YFP‐Epac‐Rluc) BRET biosensor, which changes conformation in response to cAMP levels; consequently, activation of Gα_i_ leads to a rise in BRET signal.

While the reference compounds **88**–**90** were devoid of agonist activity (Figure 4A–C), the crossover compounds **91** and **92** displayed agonist profiles similar to the initial agonist hits **16** and **15**, respectively, i. e. similar potency and partial agonist properties, meaning that the efficacy did not reach that of the full agonist **1** (Figure 4D–E). The potency of the reference endogenous agonist **1** obtained here (19 nM) (Figure 4H) was comparable to the value in the calcium assay, as were the potencies for compounds **15** and **16**. To verify that the activity was mediated by GPR183, **92** was also tested in the presence of increasing concentrations of the antagonist **5**, which potently inhibited the activity induced by both **92** and the positive control **1** (pIC_50_±SE of 8.56±0.27 and 7.86±0.16, respectively) (Figure 4F). Given the structural similarities with previously reported GPR183 antagonists, we also tested the antagonist properties of the reference compounds **88**–**90**. Compounds **89** and **90** did not inhibit the G protein activity induced by **1**, while **88** displayed weak antagonistic activity with 79 % inhibition at the highest concentration tested (10 μM). In contrast, the reference antagonist **5** resulted in almost 100 % inhibition at a lower concentration (1 μM), corresponding to an inhibitory potency of 28 nM (Figure 4G).

**Figure 4:**
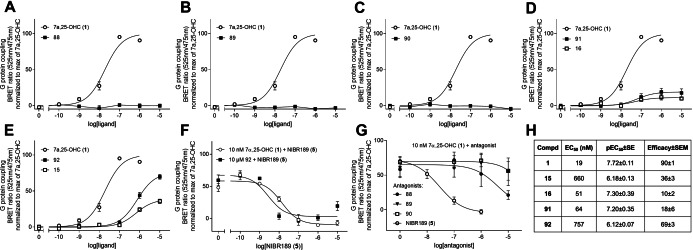
Dose‐response curves representing GPR183 G protein‐coupling upon addition of various compounds. Data is normalized to the maximum activity of the endogenous agonist **1** in each individual experiment. A–C) The reference compounds **88**–**90**. D–E) The crossover compounds **91** and **92** with initial agonist hits **15**–**16**. F) Activity of a fixed concentration of the endogenous agonist **1** and the synthesized agonist **92** with increasing amount of the antagonist **5**. G) Activity induced by 10 nM of the endogenous agonist **1** after preincubation with the reference compounds **88**–**90** or the antagonist **5**. H) Table showing potency (EC_50_), p(EC_50_) ±SE and efficacy ±SEM (determined as activity induced by 1 μM for compound **1** and by 10 μM for the other ligands) for the compounds that displayed agonist properties. All data is presented as mean ±SEM of three individual experiments.

The lack of agonist activity for the unsubstituted reference compounds (inactive **88**–**90** vs. active **11**/**16**) means that the substitution pattern on the distal rings is generally important for receptor activation. Notably, the data for the aryloxy‐based compounds (inactive **90** vs. active **15**/**91**) shows that the agonist activity is dictated by the nature of the Eastern ring system.

Comparison of the active and inactive compounds that contained the oxy‐methylene spacer indicated a pattern: aside from the 1‐naphthyl (**15**/**92**), the active compounds (**16**/**17**/**18**/**91**) are all *para*/*ortho*‐disubstituted (Figure 3, Scheme 1). This is in contrast to the comparable inactive compounds **70**–**74** (supplementary Figure S1), which contain *para*‐ and/or *meta*‐substituents. Thus, the lack of agonist activity for certain compounds seems to be linked to an inappropriate Eastern ring system, as also demonstrated by the thiophene‐containing compounds **18** (active, Figure 3) and **69** (inactive, Figure S1). Taken together, the Western ring seems to be tolerant to modifications, as a simple benzene ring (**15**/**91**) is sufficient for activity, along with other variations (**16**/**92**, **17**, **18**). On the other hand, the Eastern ring is sensitive to modifications, with minor variations in the substitution pattern having a dramatic impact on agonist activity.

To conclude, *in vitro* screening of a selection of commercially available compounds enabled us to identify novel synthetic agonists for GPR183; by supplementing these hits with a small series of in‐house compounds we were able to establish initial SAR trends. Our findings provide a basis for formulation of hypotheses regarding the structural requirements for agonist activity and suggest several avenues for further optimization aimed at potent GPR183 agonists with improved pharmacodynamic and pharmacokinetic properties. Such studies are currently underway in our laboratories.

## Conflict of interest

The authors declare no conflict of interest.

## Supporting information

As a service to our authors and readers, this journal provides supporting information supplied by the authors. Such materials are peer reviewed and may be re‐organized for online delivery, but are not copy‐edited or typeset. Technical support issues arising from supporting information (other than missing files) should be addressed to the authors.

Supporting Information
