# Supplementary material for: Discovery of GPR183 Agonists Based on an Antagonist Scaffold
Source: ChemMedChem. 2021 Aug 3;16(17):2623–7. doi: 10.1002/cmdc.202100301 (PMC8518411; doi:10.1002/cmdc.202100301)
Supplement: Supplementary file 1 — Supporting Information [file CMDC-16-2623-s001.pdf]

ChemMedChem

Supporting Information

Discovery of GPR183 Agonists Based on an Antagonist Scaffold

Viktoria M. S. Kjær, Loukas Ieremias, Viktorija Daugvilaite, Michael Lückmann,
Thomas M. Frimurer, Trond Ulven, Mette M. Rosenkilde,* and Jon Våbenø*

Supporting Information

Table of Contents

Supplementary Figure and Table

Figure S1 1-7

Table S1 8-9

Experimental Section 10-14

^{1}H and ^{13}C NMR Spectra 15-21

Figure S1 (below). Structures of the 79 screened compounds (**9**-**87**), along with the reference antagonist NIBR189 (**5**), which is shown in black with the two-atom C=C spacer highlighted in grey. The structural differences between each compound **9**-**87** and the reference compound **5** are shown in red. The compounds are divided into active (**9**-**18**) and inactive (**19**-**87**) compounds, which in turn are grouped by type of two-atom spacer (C=C, C-C, C-O, C-S, C-N).

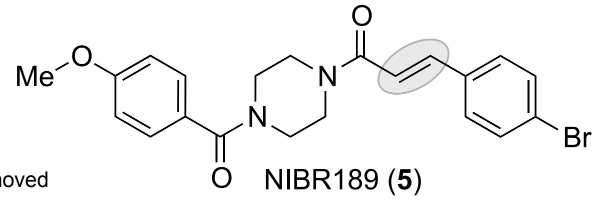

NIBR189 (**5**)

• = substituent in NIBR189 removed

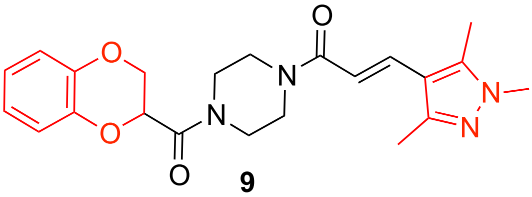

9

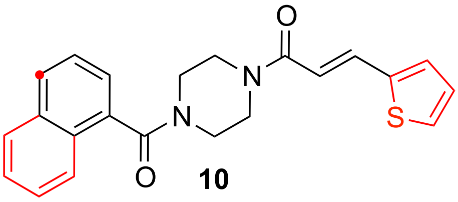

10

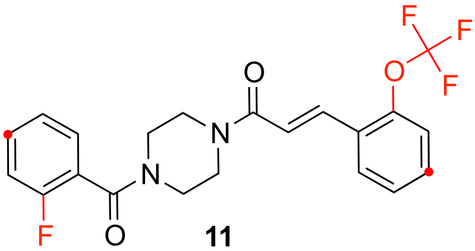

11

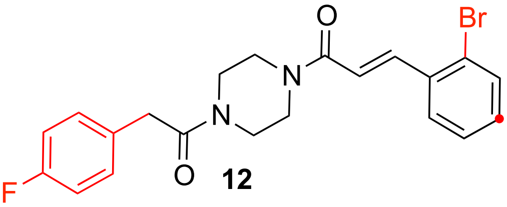

12

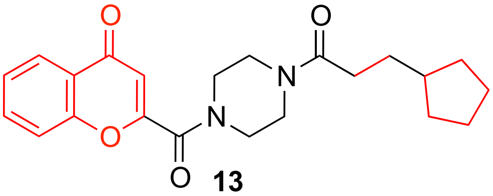

13

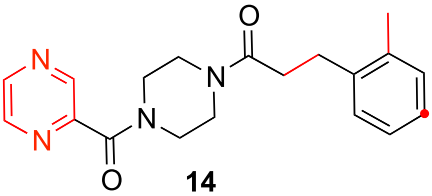

14

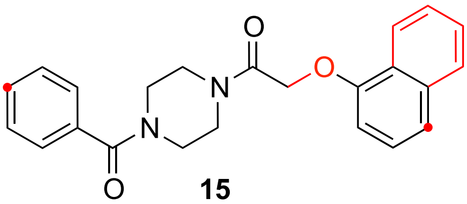

15

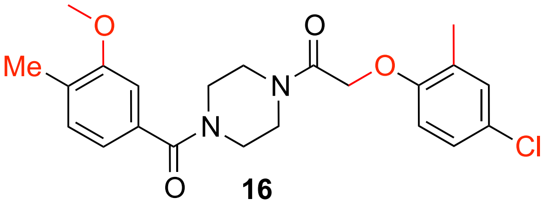

16

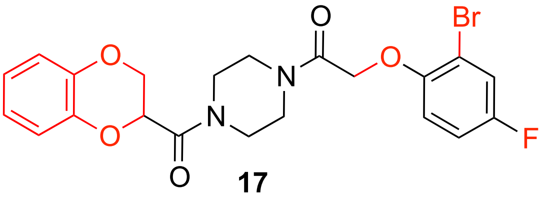

17

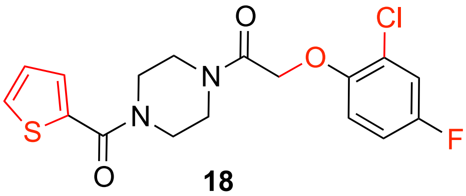

18

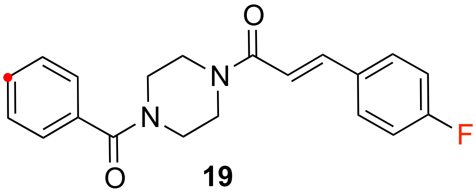

19

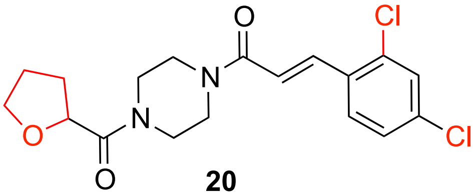

20

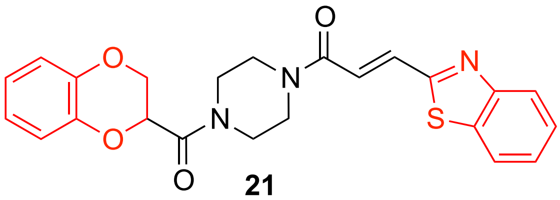

21

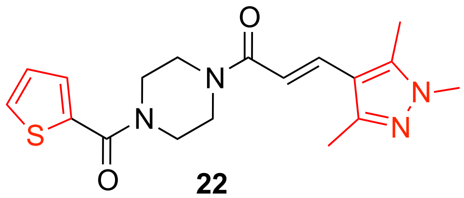

22

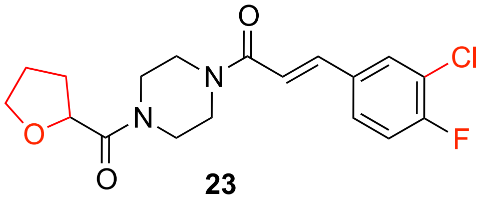

23

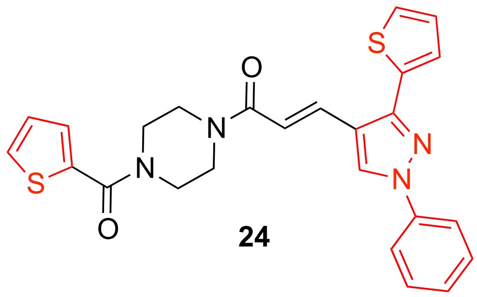

24

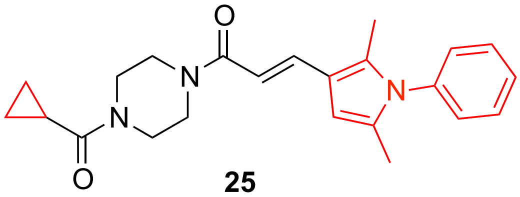

25

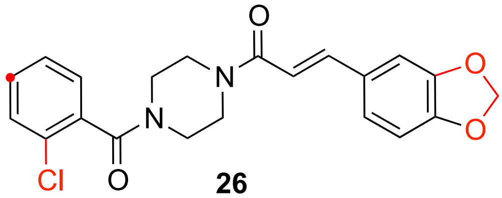

26

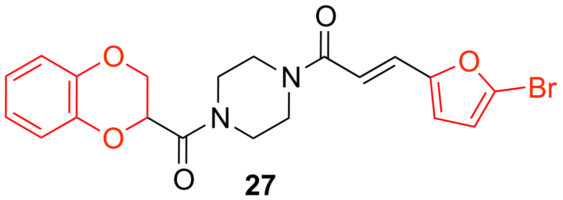

27

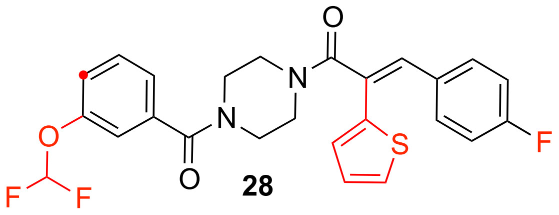

28

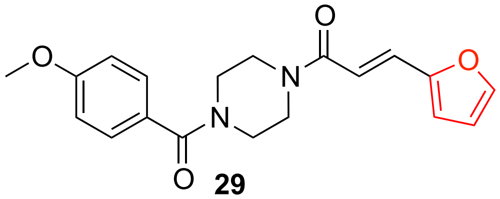

29

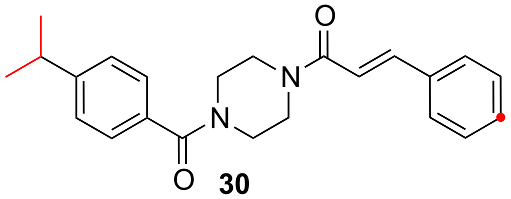

30

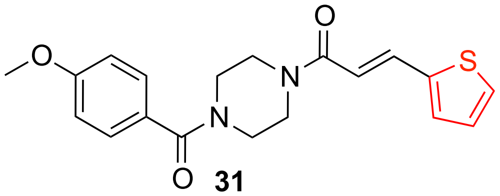

31

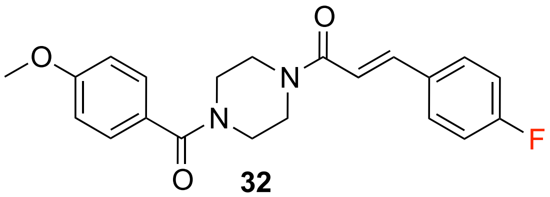

32

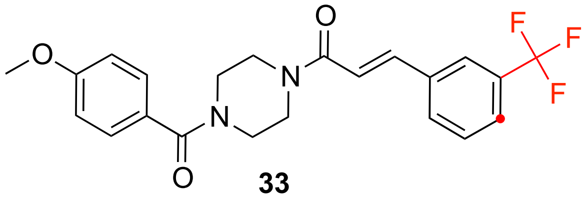

33

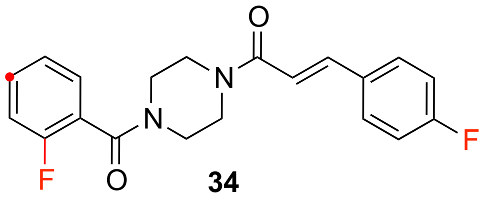

34

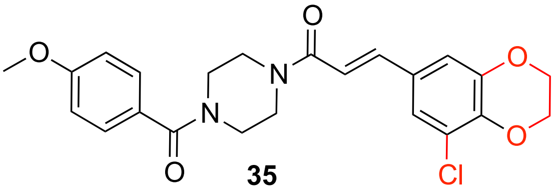

35

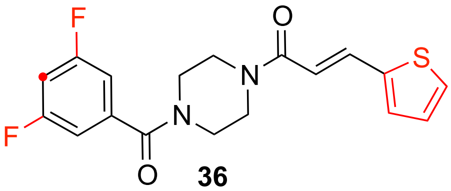

36

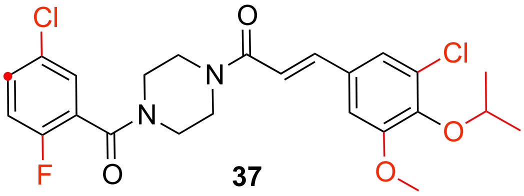

37

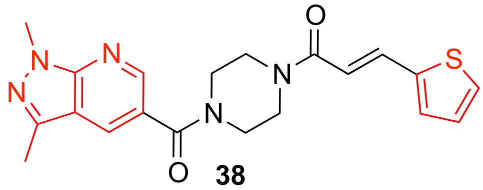

38

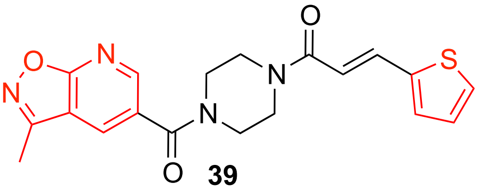

39

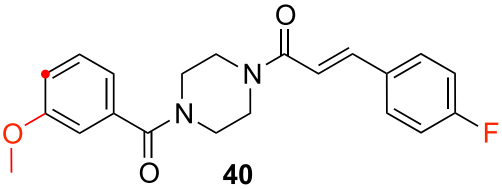

40

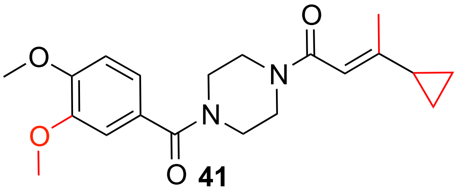

41

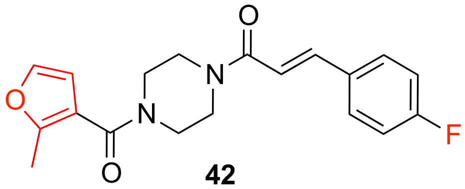

42

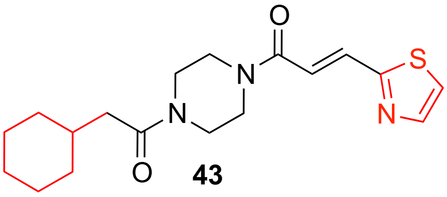

43

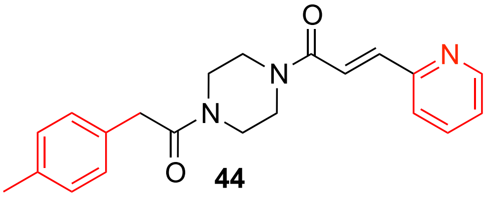

44

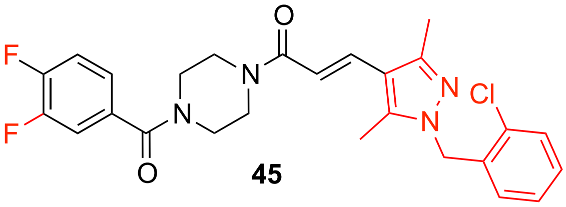

45

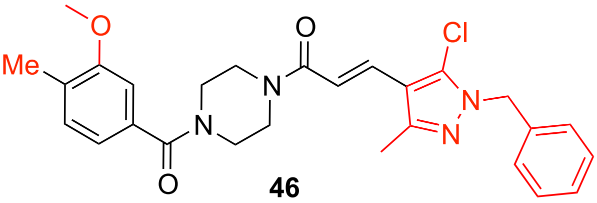

46

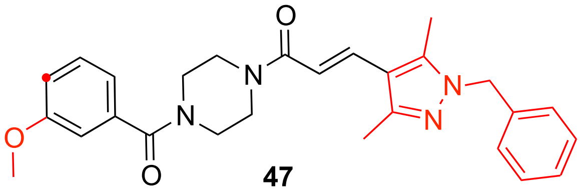

47

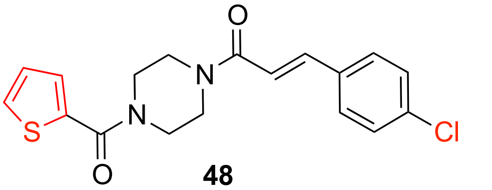

48

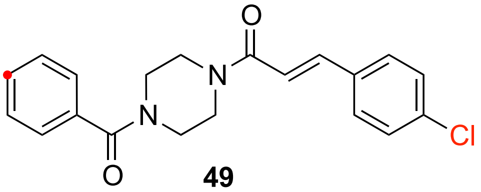

49

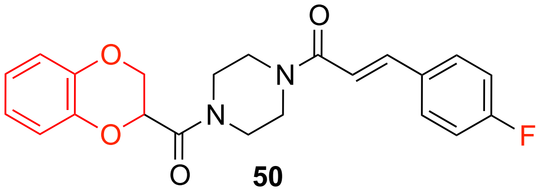

50

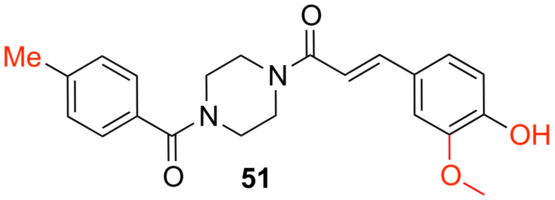

51

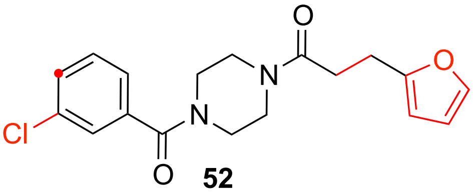

52

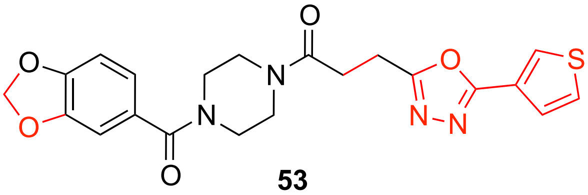

53

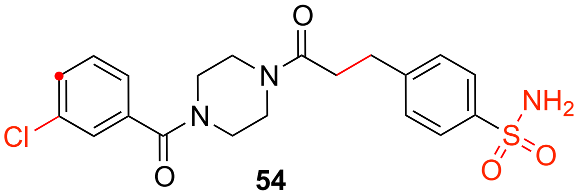

54

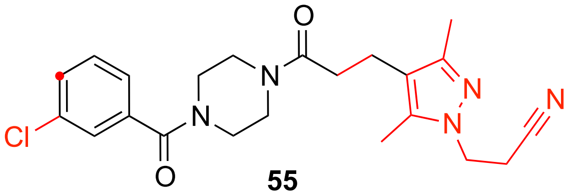

55

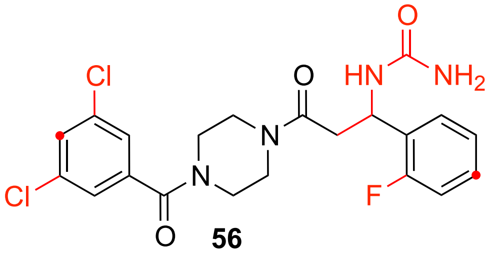

56

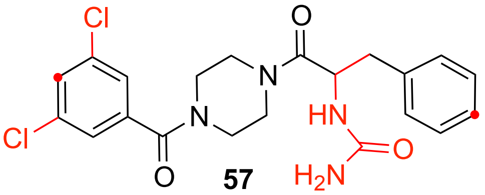

57

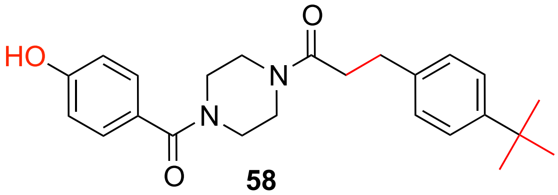

58

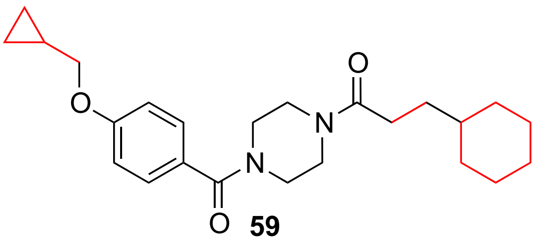

59

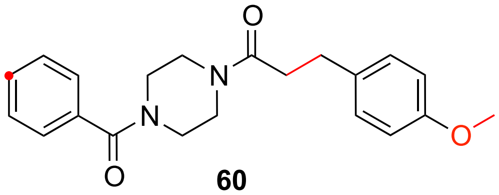

60

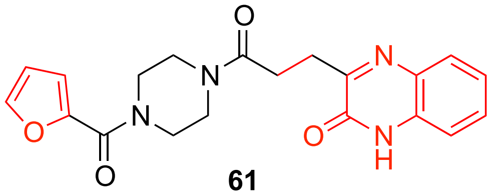

61

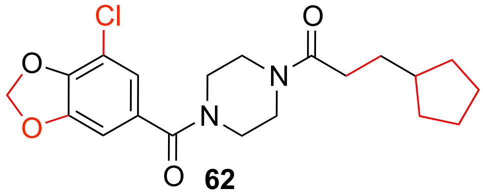
62

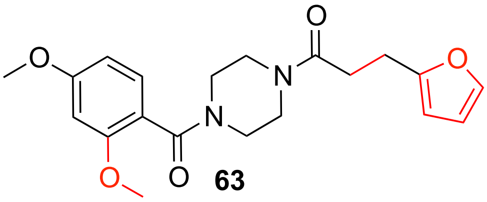
63

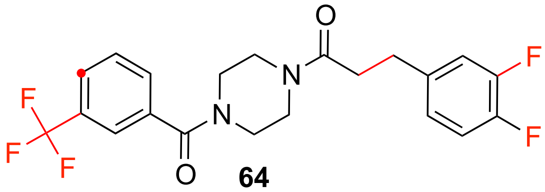
64

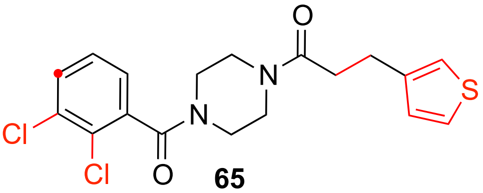
65

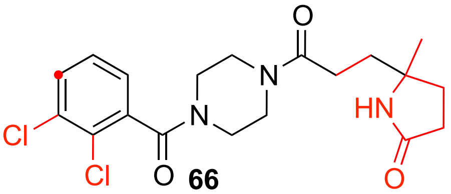
66

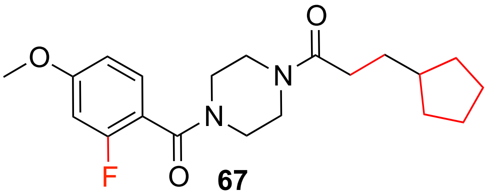
67

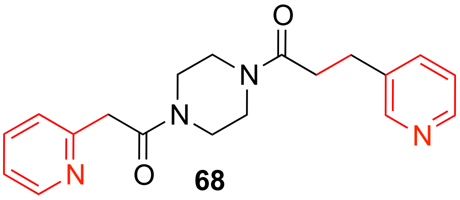
68

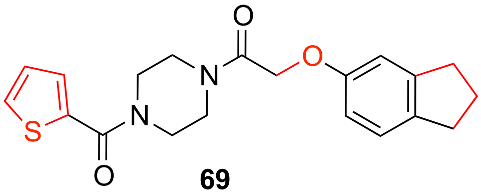
69

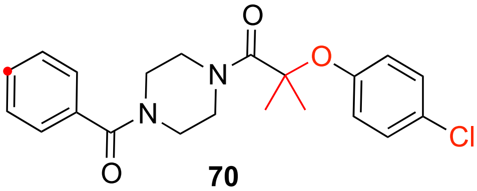
70

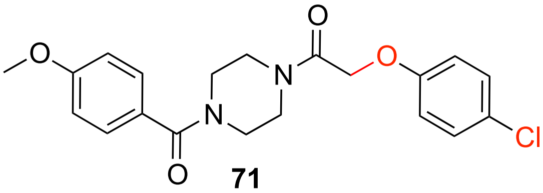
71

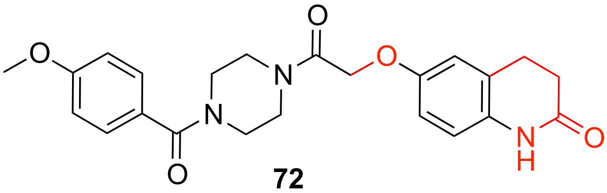
72

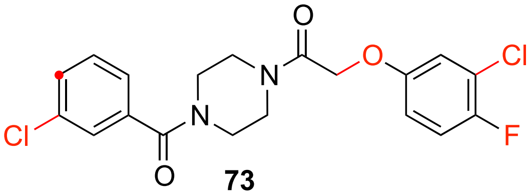
73

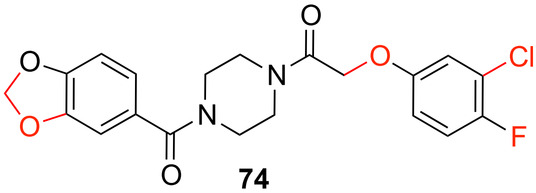
74

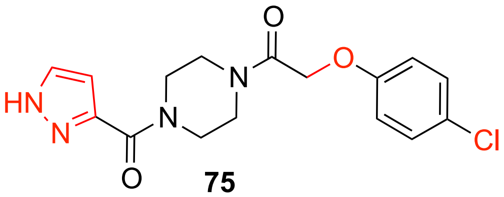

75

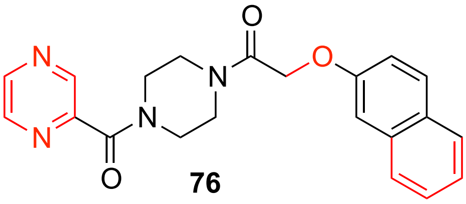

76

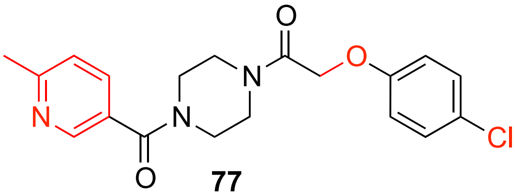

77

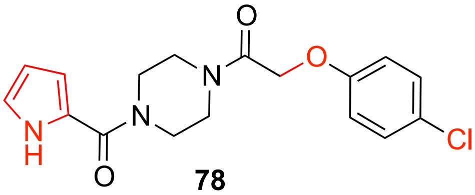

78

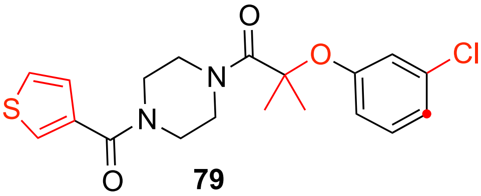

79

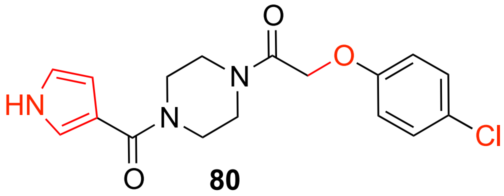

80

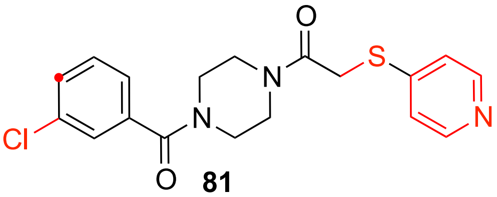

81

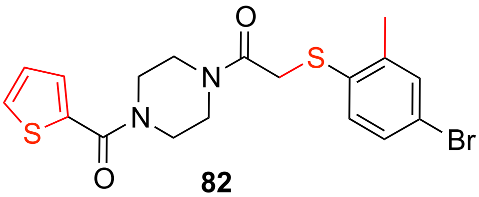

82

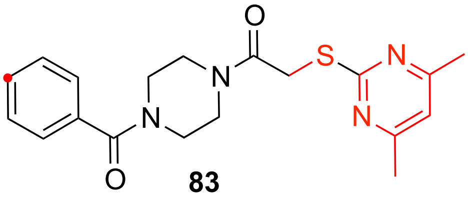

83

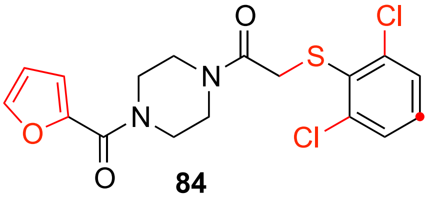

84

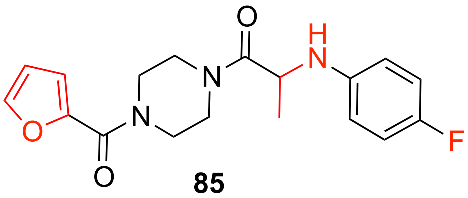

85

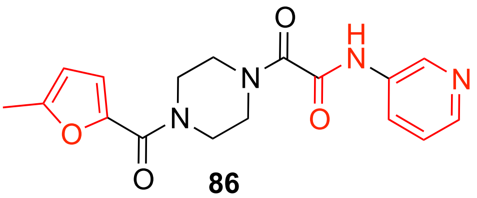

86

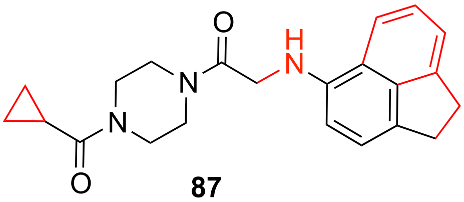

87

Table S1. SMILES and vendor IDs for the 79 screened compounds **9**-**87** (Figure S1).

No.	SMILES	ID
**9**	Cc1c(C=CC(N2CCN(CC2)C(C2COc3ccccc3O2)=O)=O)c(C)n(C)n1	Z28517117
**10**	C1CN(CCN1C(C=Cc1cccs1)=O)C(c1cccc2ccccc12)=O	Z316830628
**11**	C1CN(CCN1C(C=Cc1ccccc1OC(F)(F)F)=O)C(c1ccccc1F)=O	Z365783902
**12**	C(C(N1CCN(CC1)C(C=Cc1ccccc1[Br])=O)=O)c1ccc(cc1)F	Z765533856
**13**	C1CCC(C1)CCC(N1CCN(CC1)C(C1=CC(c2ccccc2O1)=O)=O)=O	Z333751314
**14**	Cc1ccccc1CCC(N1CCN(CC1)C(c1cnccn1)=O)=O	Z1118740455
**15**	C1CN(CCN1C(COc1cccc2ccccc12)=O)C(c1ccccc1)=O	Z26978375
**16**	Cc1ccc(cc1OC)C(N1CCN(CC1)C(COc1ccc(cc1C)[Cl])=O)=O	Z512216398
**17**	C1CN(CCN1C(COc1ccc(cc1[Br])F)=O)C(C1COc2ccccc2O1)=O	Z25080401
**18**	C1CN(CCN1C(COc1ccc(cc1[Cl])F)=O)C(c1cccs1)=O	Z62917032
**19**	C1CN(CCN1C(C=Cc1ccc(cc1)F)=O)C(c1ccccc1)=O	Z26979894
**20**	C1CC(C(N2CCN(CC2)C(C=Cc2ccc(cc2[Cl])[Cl])=O)=O)OC1	Z26966899
**21**	C1CN(CCN1C(C=Cc1nc2ccccc2s1)=O)C(C1COc2ccccc2O1)=O	Z28516715
**22**	Cc1c(C=CC(N2CCN(CC2)C(c2cccs2)=O)=O)c(C)n(C)n1	Z62915961
**23**	C1CC(C(N2CCN(CC2)C(C=Cc2ccc(c(c2)[Cl])F)=O)=O)OC1	Z26966579
**24**	C1CN(CCN1C(C=Cc1cn(c2ccccc2)nc1c1cccs1)=O)C(c1cccs1)=O	Z62914318
**25**	Cc1cc(C=CC(N2CCN(CC2)C(C2CC2)=O)=O)c(C)n1c1ccccc1	Z73339385
**26**	C1CN(CCN1C(C=Cc1ccc2c(c1)OCO2)=O)C(c1ccccc1[Cl])=O	Z52116008
**27**	C1CN(CCN1C(C=Cc1ccc(o1)[Br])=O)C(C1COc2ccccc2O1)=O	Z28517842
**28**	C1CN(CCN1C(C(=Cc1ccc(cc1)F)c1cccs1)=O)C(c1cccc(c1)OC(F)F<br>)=O	Z288716244
**29**	COc1ccc(cc1)C(N1CCN(CC1)C(C=Cc1ccco1)=O)=O	Z316644402
**30**	CC(C)c1ccc(cc1)C(N1CCN(CC1)C(C=Cc1ccccc1)=O)=O	Z316571786
**31**	COc1ccc(cc1)C(N1CCN(CC1)C(C=Cc1cccs1)=O)=O	Z316644724
**32**	COc1ccc(cc1)C(N1CCN(CC1)C(C=Cc1ccc(cc1)F)=O)=O	Z316644772
**33**	COc1ccc(cc1)C(N1CCN(CC1)C(C=Cc1cccc(c1)C(F)(F)F)=O)=O	Z316644658
**34**	C1CN(CCN1C(C=Cc1ccc(cc1)F)=O)C(c1ccccc1F)=O	Z290835098
**35**	COc1ccc(cc1)C(N1CCN(CC1)C(C=Cc1cc2c(c(c1)[Cl])OCCO2)=O)<br>=O	Z316649054
**36**	C1CN(CCN1C(C=Cc1cccs1)=O)C(c1cc(cc(c1)F)F)=O	Z421820312
**37**	CC(C)Oc1c(cc(C=CC(N2CCN(CC2)C(c2cc(ccc2F)[Cl])=O)=O)cc1[<br>Cl])OC	Z423073330
**38**	Cc1c2cc(cnc2n(C)n1)C(N1CCN(CC1)C(C=Cc1cccs1)=O)=O	Z431696316
**39**	Cc1c2cc(cnc2on1)C(N1CCN(CC1)C(C=Cc1cccs1)=O)=O	Z431696226
**40**	COc1cccc(c1)C(N1CCN(CC1)C(C=Cc1ccc(cc1)F)=O)=O	Z316629248
**41**	CC(=CC(N1CCN(CC1)C(c1ccc(c(c1)OC)OC)=O)=O)C1CC1	Z735797608
**42**	Cc1c(cco1)C(N1CCN(CC1)C(C=Cc1ccc(cc1)F)=O)=O	Z776465636
**43**	C1CCC(CC1)CC(N1CCN(CC1)C(C=Cc1nccs1)=O)=O	Z996754552
**44**	Cc1ccc(CC(N2CCN(CC2)C(C=Cc2ccccn2)=O)=O)cc1	Z997313718
**45**	Cc1c(C=CC(N2CCN(CC2)C(c2ccc(c(c2)F)F)=O)=O)c(C)n(Cc2cccc<br>c2[Cl])n1	Z346556286
**46**	Cc1ccc(cc1OC)C(N1CCN(CC1)C(C=Cc1c(C)nn(Cc2ccccc2)c1[Cl])<br>=O)=O	Z512206574
**47**	Cc1ccc(Cn2c(C)c(C=CC(N3CCN(CC3)C(c3cccc(c3)OC)=O)=O)c(C<br>)n2)cc1	Z316631718

**48**	C1CN(CCN1C(C=Cc1ccc(cc1)[Cl])=O)C(c1cccs1)=O	Z62913853
**49**	C1CN(CCN1C(C=Cc1ccc(cc1)[Cl])=O)C(c1ccccc1)=O	Z26979730
**50**	C1CN(CCN1C(C=Cc1ccc(cc1)F)=O)C(C1COc2ccccc2O1)=O	Z28518001
**51**	Cc1ccc(cc1)C(N1CCN(CC1)C(C=Cc1ccc(c(c1)OC)O)=O)=O	Z1690242033
**52**	C(Cc1ccco1)C(N1CCN(CC1)C(c1cccc(c1)[Cl])=O)=O	Z289142814
**53**	C(Cc1nnc(c2ccsc2)o1)C(N1CCN(CC1)C(c1ccc2c(c1)OCO2)=O)=O	Z296634412
**54**	C(Cc1ccc(cc1)S(N)(=O)=O)C(N1CCN(CC1)C(c1cccc(c1)[Cl])=O)=O	Z288399946
**55**	Cc1c(CCC(N2CCN(CC2)C(c2cccc(c2)[Cl])=O)=O)c(C)n(CCC#N)n1	Z301785596
**56**	C(C(c1ccccc1F)NC(N)=O)C(N1CCN(CC1)C(c1cc(cc(c1)[Cl])[Cl])=O)=O	Z337225338
**57**	C(C(C(N1CCN(CC1)C(c1cc(cc(c1)[Cl])[Cl])=O)=O)NC(N)=O)c1ccccc1	Z306744400
**58**	CC(C)(C)c1ccc(CCC(N2CCN(CC2)C(c2ccc(cc2)O)=O)=O)cc1	Z358336906
**59**	C1CCC(CC1)CCC(N1CCN(CC1)C(c1ccc(cc1)OCC1CC1)=O)=O	Z809085068
**60**	COc1ccc(CCC(N2CCN(CC2)C(c2ccccc2)=O)=O)cc1	Z26978521
**61**	C(CC(N1CCN(CC1)C(c1ccco1)=O)=O)C1C(Nc2ccccc2N=1)=O	Z30622437
**62**	C1CCC(C1)CCC(N1CCN(CC1)C(c1cc2c(c(c1)[Cl])OCO2)=O)=O	Z333752092
**63**	COc1ccc(C(N2CCN(CC2)C(CCc2ccco2)=O)=O)c(c1)OC	Z740641210
**64**	C(Cc1ccc(c(c1)F)F)C(N1CCN(CC1)C(c1cccc(c1)C(F)(F)F)=O)=O	Z436997324
**65**	C(Cc1ccsc1)C(N1CCN(CC1)C(c1cccc(c1[Cl])[Cl])=O)=O	Z437697060
**66**	CC1(CCC(N1)=O)CCC(N1CCN(CC1)C(c1cccc(c1[Cl])[Cl])=O)=O	Z1575245888
**67**	COc1ccc(C(N2CCN(CC2)C(CCC2CCCC2)=O)=O)c(c1)F	Z333752288
**68**	C(Cc1cccnc1)C(N1CCN(CC1)C(Cc1ccccn1)=O)=O	Z1116824951
**69**	C1Cc2ccc(cc2C1)OCC(N1CCN(CC1)C(c1cccs1)=O)=O	Z62915291
**70**	CC(C)(C(N1CCN(CC1)C(c1ccccc1)=O)=O)Oc1ccc(cc1)[Cl]	Z26979831
**71**	COc1ccc(cc1)C(N1CCN(CC1)C(COc1ccc(cc1)[Cl])=O)=O	Z274398464
**72**	COc1ccc(cc1)C(N1CCN(CC1)C(COc1ccc2c(CCC(N2)=O)c1)=O)=O	Z318384868
**73**	C1CN(CCN1C(COc1ccc(c(c1)[Cl])F)=O)C(c1cccc(c1)[Cl])=O	Z300435064
**74**	C1CN(CCN1C(COc1ccc(c(c1)[Cl])F)=O)C(c1ccc2c(c1)OCO2)=O	Z300438840
**75**	C1CN(CCN1C(COc1ccc(cc1)[Cl])=O)C(c1cc[nH]n1)=O	Z738817178
**76**	C1CN(CCN1C(COc1ccc2ccccc2c1)=O)C(c1cnccn1)=O	Z994323478
**77**	Cc1ccc(cn1)C(N1CCN(CC1)C(COc1ccc(cc1)[Cl])=O)=O	Z730565118
**78**	C1CN(CCN1C(COc1ccc(cc1)[Cl])=O)C(c1ccc[nH]1)=O	Z757665966
**79**	CC(C)(C(N1CCN(CC1)C(c1ccsc1)=O)=O)Oc1cccc(c1)[Cl]	Z1188710439
**80**	C1CN(CCN1C(COc1ccc(cc1)[Cl])=O)C(c1cc[nH]c1)=O	Z1167549896
**81**	C1CN(CCN1C(CSc1ccncc1)=O)C(c1cccc(c1)[Cl])=O	Z289143168
**82**	Cc1cc(ccc1SCC(N1CCN(CC1)C(c1cccs1)=O)=O)[Br]	Z62914144
**83**	Cc1cc(C)nc(n1)SCC(N1CCN(CC1)C(c1ccccc1)=O)=O	Z31939947
**84**	C1CN(CCN1C(CSc1c(cccc1[Cl])[Cl])=O)C(c1ccco1)=O	Z17831697
**85**	CC(C(N1CCN(CC1)C(c1ccco1)=O)=O)Nc1ccc(cc1)F	Z104599044
**86**	Cc1ccc(C(N2CCN(CC2)C(C(Nc2cccnc2)=O)=O)=O)o1	Z1019851870
**87**	C1Cc2ccc(c3cccc1c23)NCC(N1CCN(CC1)C(C1CC1)=O)=O	Z739341864

Experimental Section

In silico substructure screen

The Enamine Screening Collection, containing 2.776.742 in stock available compounds from Enamine Ltd. (Kiev, Ukraine) was used in a substructure screen using the Markush structure **7** shown in Figure 1B as query. SurflexSim (version 2.5) was used with the options –pscreen to conduct the screening. The results were filtered based on chemical criteria (molecular weight < 500 g/mol, number of rotatable bonds < 10, logP < 5) and clustered based on a T_c threshold of 0.2. One compound was selected from each cluster. Finally, 79 compounds were manually selected for experimental screening.

Tissue culture and transfection

Chinese hamster ovary (CHO-K1) cells (ATCC® CCL-61) were grown at 5% CO_2 and 37 °C in RPMI 1640 supplemented with 10% (v/v) FBS, 2 mM GlutaMAX (Gibco), 180 units ml^{-1} penicillin and 45 µg ml^{-1} streptomycin and split once confluent (around every 3rd day). Transient transfection of CHO-K1 cells was performed by lipofection using Lipofectamine 2000 (Invitrogen) according to the manufacturer’s instructions. Lipofectamine™ 2000 reagent and Opti-MEM were purchased from Invitrogen (Glasgow, UK). Human GPR183 contained an N-terminal M1-FLAG and was inserted into a modified pcDNA3 vector which contained an upstream sequence encoding a hemagglutinin signal peptide fused to the FLAG tag. The promiscuous chimeric G protein GαΔ6qi4myr (abbreviated Gqi4myr) was kindly provided by Evi Kostenis (Rheinische Friedrich-Wilhelm University, Bonn, Germany) and the CAMYEL sensor was kindly provided by Jonathan Javitch (Columbia University, New York, US). EC_{50} and pEC_{50} values determined in Prism using non-linear curve fit.

BRET cAMP

2 days prior to assay, CHO-K1 cells were seeded 500.000/well in 6-well plates. The following day cells were transfected using 6 µl Lipofectamine, 160 ng receptor DNA and 840 ng CAMYEL DNA in each well. On assay day, cells from each well were harvested in 3 ml PBS with 5mM glucose and added to a 96-well plate, 84 µl in each. Just before ligand addition, 10 µl coelenterazine (5 µM in well) was added followed by 1 µl 100X ligand in DMSO. 5 minutes post ligand addition 5 µl forskolin (10 µM in well) was added and at 40 min post ligand addition, luminescence (475 nm) and fluorescence (525 nm) were measured using the EnVision plate reader (PerkinElmer). For the testing of antagonism, the antagonist (100X in DMSO) was added 10 min prior to ligand addition. EC_{50} and pEC_{50} values were determined in Prism using non-linear curve fit.

Ca^{2+} release assay

CHO-K1 cells were seeded in 96-well clear bottom black plates at a density of 2 x 10^4 cells per well. The following day the cells were transfected with GPR183 (30 ng/well) and Gqi4myr (10 ng/well). 24 h after transfection, cells were washed once with 100 µl of HBSS pre-warmed to 37 °C supplemented with 20 mM HEPES (Invitrogen), 1 mM $CaCl_2$, 1 mM $MgCl_2$, and 0.7 mg ml^{-1} probenicid (Life Technologies, Thermo Fisher Scientific)). Afterwards, cells were incubated for 60 min at 37 °C and 5% CO_2 with 50 µl of pre-warmed loading buffer per well (wash buffer supplemented with 0.2% (v/v) Fluo-4 (Life Technologies, Thermo Fisher Scientific)) and covered from light. After incubation, cells were washed twice with 75 µl of pre- warmed wash buffer, and 100 µl of 37 °C pre-warmed cell medium was added. Ligands

were added and plates measured using a FlexStation 3 Multi-Mode Microplate Reader (Molecular Devices) with excitation and emission wavelengths of 485 and 525 nm, respectively. Determinations were made in duplicates.

Chemistry – General

All reagents were of commercial grade and were used as received without further purification. Anhydrous reactions were carried out in flame-dried glassware under argon atmosphere. Dry chromatographic grade dichloromethane (DCM), tetrahydrofurane (THF) and dimethylformamide (DMF) were obtained from a Waters SG solvent purification system. Thin layer chromatography (TLC) Silica gel 60 F254, Merck pre-coated plates were used, visualized under UV light (254 or 365nm), developed in the system stated for each compound. Flash chromatography of compounds was performed using silica gel 60 (40-64 µm), where loading of the compounds was done after dry mixing with Celite.

Nuclear Magnetic Resonance (NMR) spectra were recorded on 400 or 600 MHz Bruker instruments (^{1}H NMRs were obtained at 400 or 600 MHz and ^{13}C NMRs were obtained at 100 or 151MHz). The obtained FID files were processed with Mnova 14 software. Spectra are calibrated relative to residual solvent peaks. Multiplet patterns are designated the following abbreviations, or combinations of these: m – multiplet, s – singlet, d – doublet, t – triplet, q – quartet, p – pentet, h – sextet.

Mass spectrometry (MS) was performed on an Aquity UPLC instrument connected to an Aquity TUV detector and an Aquity QDa detector. Gradient: 100% A to 100% B over 5 min. Mobile phase A: MeCN 5%, formic acid 0.1% in water, Mobile Phase B: MeCN 99.9%, formic acid 0.1%. Flow rate: 0.5 mL/min. Samples used in this system were dissolved in 1:1 MeCN, water or MeOH.

Analytical High Performance Liquid Chromatography (HPLC) was performed on a Dionex UltiMate HPLC system consisting of an LPG-3400A pump (1 mL/min), a WPS-3000L autosampler and a DAD-3000D diode array detector (210, 254, 290, 365 nm), using a Gemini-NX C18 column (4.6 × 250 mm, 3 µm, 110 Å); Mobile phase A: Trifluoroacetic acid (TFA) 0.1% in water. Mobile phase B: Water 10%, TFA 0.1% in MeCN. Data were acquired and processed using the Chromeleon Software v. 6.80. Method used for analysis: Gradient 0-15 min, 50-100% Mobile phase B.

Mass analysis by matrix-assisted laser desorption/ionization high-resolution mass spectrometry (MALDI-HRMS) was performed on a QExactive Orbitrap mass spectrometer (Thermo Scientific, Bremen, Germany) equipped with a SMALDI5 ion source (TransMIT GmbH, Giessen, Germany). The sample was analyzed in the positive ion mode using a peak from the DHB matrix for internal mass calibration whereby a mass accuracy of 2 ppm or better was achieved. The samples (1 mg) were mixed with a 20 mg/mL solution of 2,5-dihydroxybenzoic acid in MeOH as MALDI matrix, 2 µL of the mixture was deposited on a 30-well glass plate (Electron Microscopy Sciences, Hatfield, PA, USA) and analysis followed upon evaporation.

Chemistry - Synthesis

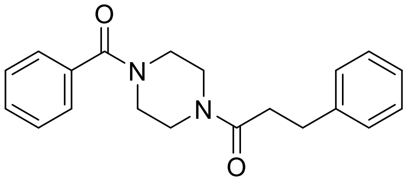

1-(4-Benzoylpiperazin-1-yl)-3-phenylpropan-1-one (88). A flame dried round bottom flask was sequentially charged with *N*-benzoylpiperazine (44.8 mg, 0.236 mmol, 1 eq) DCM (4.5 mL), and triethylamine (TEA) (0.05 mL, 0.359 mmol, 1.5 eq). 3-Phenylpropanoyl chloride (0.07 mL, 0.471 mmol, 2 eq) was added at room temperature (rt). After 24 h, the reaction mixture was washed with aq. HCl solution (1 M, 5 mL), followed by aq. NaOH solution (1 M, 5 mL) and brine (5 mL). The organic phase was dried over Na_2SO_4, filtered and the solvent was evaporated under reduced pressure. The residue was purified by silica gel column chromatography (65-75% EtOAc in heptane) to afford **89** (68.0 mg, 90%) as colorless oil. Rf = 0.19 (EtOAc: petroleum ether (PE), 5:5). ^{1}H NMR (400 MHz, $CDCl_3$) δ 7.45 – 7.35 (m, 5H), 7.32 – 7.18 (m, 5H), 3.81 – 3.20 (m, 8H), 2.98 (t, *J* = 7.7 Hz, 2H), 2.64 (t, *J* = 7.5 Hz, 2H). ^{13}C NMR (101 MHz, $CDCl_3$) δ 171.0, 170.6, 141.0, 135.2, 130.2, 128.7, 128.7, 128.6, 127.1, 126.4, 45.5, 41.8, 35.0, 31.6. HRMS (MALDI): m/z calcd for $C_{20}H_{23}N_2O_2$ (M+H$^+$) 323.1754, found 323.1752. HPLC: t_R = 5.18 min, Purity: 98.41%.

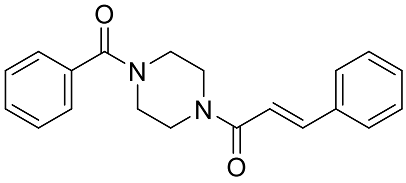

(*E*)-1-(4-benzoylpiperazin-1-yl)-3-phenylprop-2-en-1-one (89). The compound was synthesized as described for **88**, using cinnamoyl chloride (78.8 mg, 0.473 mmol) and purified by silica gel column chromatography (60-75% EtOAc in heptane) to afford 64.7 mg (84%) white solid. Rf = 0.33 (EtOAc:PE, 8:2). ^{1}H NMR (400 MHz, $CDCl_3$) δ 7.71 (d, *J* = 15.4 Hz, 1H), 7.61 – 7.48 (m, 2H), 7.49 – 7.26 (m, 8H), 6.86 (d, *J* = 15.3 Hz, 1H), 3.63 (d, *J* = 78.5 Hz, 8H). ^{13}C NMR (101 MHz, $CDCl_3$) δ 170.8, 165.8, 143.8, 135.3, 135.1, 130.2, 130.0, 129.0, 128.8, 128.0, 127.2, 116.5, 45.8, 42.2. HRMS (MALDI): m/z calcd for $C_{20}H_{21}N_2O_2$ (M+H$^+$) 321.1597, found 321.1596. HPLC: t_R = 5.33 min, Purity: 98.73%.

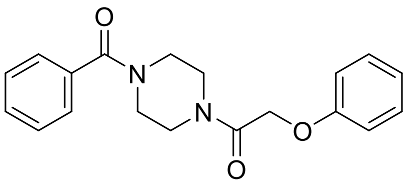

1-(4-Benzoylpiperazin-1-yl)-2-phenoxyethan-1-one (90). To a solution of 2-phenoxyacetic acid (30 mg, 0.197 mmol, 1 eq) and 1-hydroxybenzotriazole (HOBt) (51.0 mg, 0.377 mmol, 1.9 eq) in dry DMF (1 mL), ethylcarbodiimide (EDC) HCl (41.6 mg, 0.217 mmol, 1.1 eq) and phenyl(piperazin-1-yl)methanone (41.3 mg, 0.217 mmol, 1.1 eq) were added at 0 °C. After 10 min DIPEA (0.04 mL, 0.230 mmol, 1.2 eq) was added at 0 °C, the ice bath was removed and the reaction mixture was stirred at rt. After 24 h EtOAc (10 mL) was added and the organic phase was washed with aq. HCl solution (1 M, 2 X 5 mL), followed by aq. NaOH solution (1 M, 5 mL) and brine (5 mL). The organic layer was dried over Na_2SO_4, filtered and the solvent was

evaporated under reduced pressure to afford **90** (59 mg, 92%) as white solid. Rf = 0.34 (EtOAc:PE, 8:2). ^{1}H NMR (400 MHz, $CDCl_3$) δ 8.00 (s, 0H), 7.46 – 7.35 (m, 5H), 7.28 (t, *J* = 7.8 Hz, 2H), 7.06 – 6.83 (m, 3H), 4.70 (s, 2H), 3.89 – 3.32 (m, 8H). ^{13}C NMR (101 MHz, $CDCl_3$) δ 170.7, 166.9, 157.6, 135.2, 130.2, 129.8, 128.7, 127.1, 122.0, 114.6, 67.9, 45.6, 42.3. HRMS (MALDI): m/z calcd for $C_{19}H_{21}N_2O_3$ (M+H$^+$) 325.1546, found 325.1545. HPLC: t_R = 4.70 min, Purity: 99.99%.

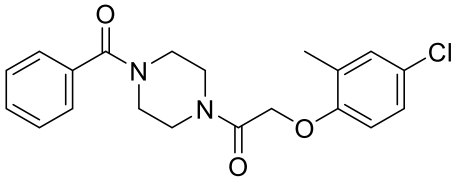

1-(4-Benzoylpiperazin-1-yl)-2-(4-chloro-2-methylphenoxy)ethan-1-one (91). The compound was synthesized as described for **90**, using 2-(4-chloro-2-methylphenoxy)acetic acid (38.5 mg, 0.192 mmol) to afford 69.1 mg (97%) white solid. Rf = 0.38 (EtOAc:PE, 8:2). ^{1}H NMR (400 MHz, $CDCl_3$) δ 7.45 – 7.37 (m, 5H), 7.19 – 7.04 (m, 2H), 6.77 (d, *J* = 8.6 Hz, 1H), 4.71 (s, 2H), 3.86 – 3.37 (m, 8H), 2.21 (s, 3H). ^{13}C NMR (101 MHz, $CDCl_3$) δ 170.7, 166.7, 154.5, 135.1, 131.0, 130.3, 128.8, 128.5, 127.2, 126.8, 126.4, 112.2, 68.2, 45.6, 42.4, 16.3. HRMS (MALDI): m/z calcd for $C_{20}H_{22}ClN_2O_3$ (M+H$^+$) 373.1313, found 373.1310. HPLC: t_R = 6.38 min, Purity: 96.30%.

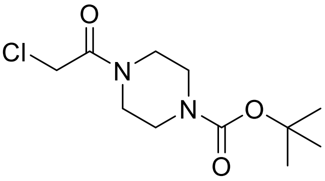

***tert*-Butyl 4-(2-chloroacetyl)piperazine-1-carboxylate (95).** A flame dried round bottom flask was sequentially charged with *tert*-butyl piperazine-1-carboxylate (150 mg, 0.805 mmol, 1 eq), DCM (14.5 mL), and TEA (0.17 mL, 1.22 mmol, 1.5 eq). Then 2-chloroacetyl chloride (0.13 mL, 1.63 mmol, 2 eq) was added dropwise at 0 ^{o}C and the mixture was allowed to stir at rt. After 24 h, the reaction mixture was washed with aq. NaOH solution (1 M, 10 mL) and brine (10 mL). The organic fraction was dried with anhydrous Na_2SO_4, filtered and the solvent was evaporated under reduced pressure to afford **95** as yellow oil (210 mg, 99%) which was used in the next reaction without further purification. Rf = 0.57 (EtOAc:PE, 8:2). ^{1}H NMR (400 MHz, $CDCl_3$) δ 4.05 (s, 2H), 3.60 – 3.53 (m, 2H), 3.47 (s, 4H), 3.43 – 3.37 (m, 2H), 1.44 (s, 9H). ^{13}C NMR (101 MHz, $CDCl_3$) δ 165.4, 154.5, 80.5, 46.2, 42.1, 40.9, 28.4.

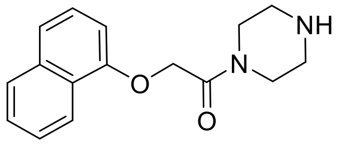

2-(Naphthalen-1-yloxy)-1-(piperazin-1-yl)ethan-1-one (96). A round bottom flask was charged with tert-butyl 4-(2-chloroacetyl)piperazine-1-carboxylate (210.0 mg, 0.799 mmol, 1 eq), naphthalen-1-ol (172.9 mg, 1.20 mmol, 1.5 eq), Cs_2CO_3 (520.9 mg, 1.60 mmol, 2 eq), KI (6.6 mg, 0.040 mmol, 0.05 eq), then acetone (4 mL) was added and the temperature was set at 35 ^{o}C. After 24 h the solvent was evaporated, and the residue was taken up in EtOAc (20 mL). The organic solution was washed with aq. NaOH solution (1 M, 2 X 10 mL) and brine (10 mL). The organic phase was dried over Na_2SO_4, filtered and the solvent was evaporated under reduced pressure. The residue was purified by silica gel column chromatography (5-40%

EtOAc in heptane) to afford an orange oil (244 mg, 82%), which was subjected directly to the next reaction (R_f = 0.62 (EtOAC:PE, 7:3)).

In a solution of tert-butyl 4-(2-(naphthalen-1-yloxy)acetyl)piperazine-1-carboxylate (244.0 mg, 0.659 mmol) in DCM (1 mL), TFA (1 mL) was added and the mixture was stirred at rt for 1 h. Then water (10 mL) and DCM (10 mL) was added and the reaction was neutralized by addition of solid $NaHCO_3$. After neutralization, the mixture was transferred to a separation funnel, the phases were separated, and the organic phase was washed with aq. sat. $NaHCO_3$ solution (2 X 10 mL). The organic phase was dried over Na_2SO_4, filtered and the solvent was evaporated under reduced pressure to afford **96** as orange oil (132 mg, 74%), which was used in the next reaction without further purification. ^1H NMR (400 MHz, $CDCl_3$) δ 8.28 – 8.20 (m, 1H), 7.85 – 7.76 (m, 1H), 7.54 – 7.43 (m, 3H), 7.36 (t, *J* = 8.0 Hz, 1H), 6.88 (d, *J* = 7.7 Hz, 1H), 4.87 (s, 2H), 3.70 – 3.56 (m, 4H), 2.92 – 2.74 (m, 4H), 2.27 (s, 1H). ^{13}C NMR (101 MHz, $CDCl_3$) δ 166.6, 153.5, 134.7, 127.7, 126.7, 125.9, 125.6, 125.5, 121.8, 121.4, 105.3, 68.2, 46.8, 46.4, 45.9, 43.2.

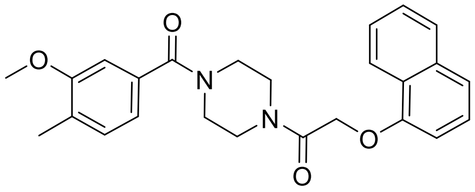

1-(4-(3-Methoxy-4-methylbenzoyl)piperazin-1-yl)-2-(naphthalen-1-yloxy)ethan-1-one (92). The compound was synthesized as described for **90**, using 3-methoxy-4-methylbenzoic acid (73.8 mg, 0.444 mmol) and purified by silica gel column chromatography (70-90% EtOAc in heptane) to afford 178.5 mg (96%) white foam. Rf = 0.22 (EtOAc:PE, 8:2). ^1H NMR (400 MHz, $CDCl_3$) δ 8.28 – 8.11 (m, 1H), 7.85 – 7.79 (m, 1H), 7.54 – 7.45 (m, 3H), 7.37 (t, *J* = 8.0 Hz, 1H), 7.12 (d, *J* = 7.5 Hz, 1H), 6.91 (d, *J* = 7.6 Hz, 1H), 6.85 (s, 1H), 6.81 (d, *J* = 7.4 Hz, 1H), 4.92 (s, 2H), 3.81 (s, 3H), 3.79 – 3.22 (m, 8H), 2.22 (s, 3H). ^{13}C NMR (101 MHz, $CDCl_3$) δ 170.8, 166.9, 158.0, 153.2, 134.7, 133.6, 130.5, 129.3, 127.8, 126.8, 125.9, 125.8, 125.4, 121.7, 121.5, 118.9, 109.1, 105.3, 68.3, 55.5, 45.8, 42.5, 16.3. HRMS (MALDI): m/z calcd for $C_{25}H_{27}N_2O_4$ ($M+H^+$) 419.1965, found 419.1963. HPLC: t_R = 7.33 min, Purity: 99.65%.

^{1}H and ^{13}C NMR Spectra

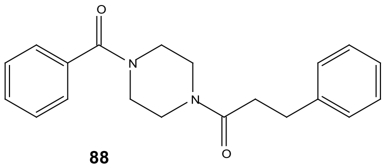

88

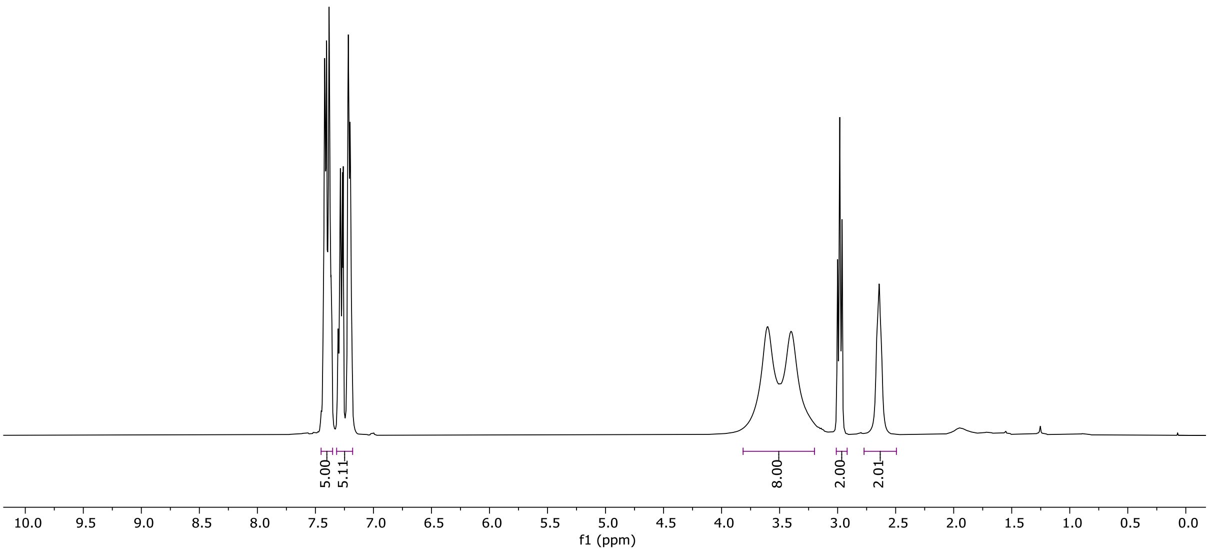

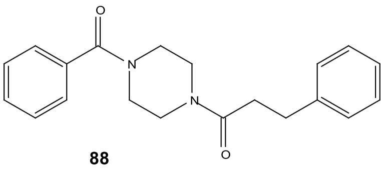

88

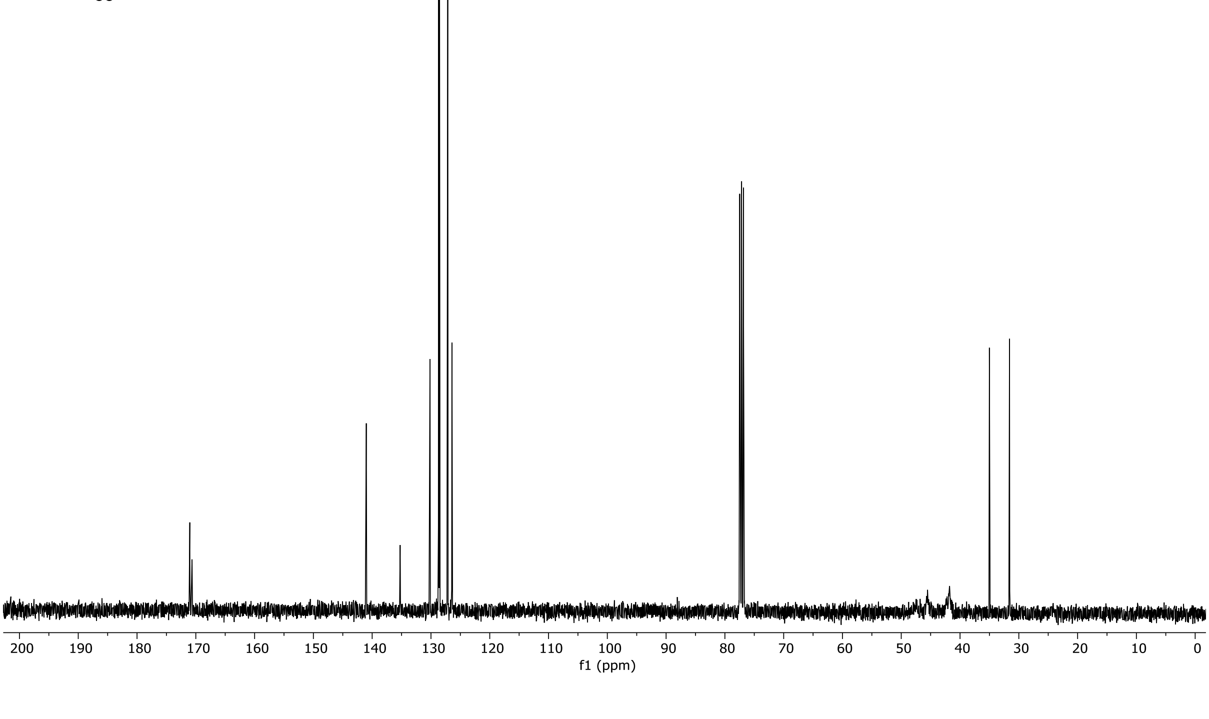

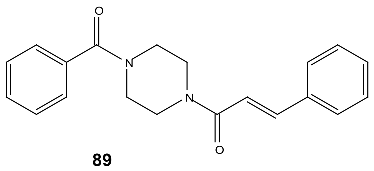

89

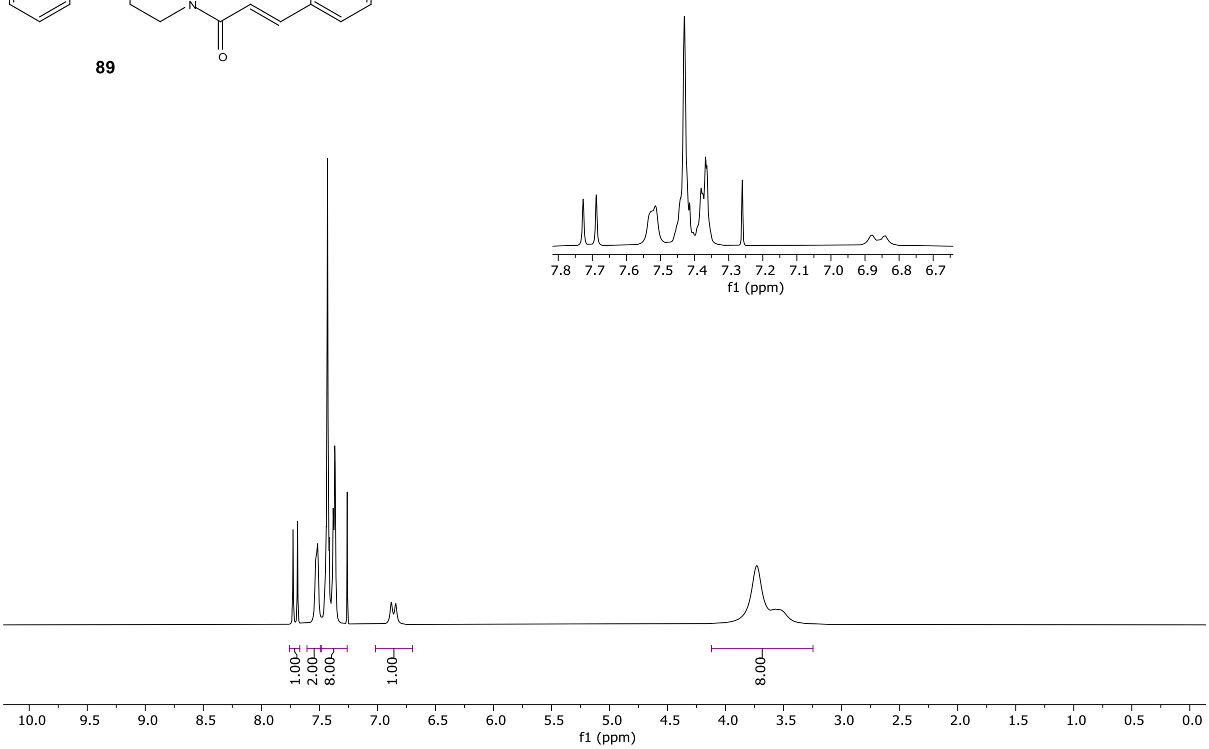

7.8 7.7 7.6 7.5 7.4 7.3 7.2 7.1 7.0 6.9 6.8 6.7
f1 (ppm)
1.00
2.00
8.00
1.00
8.00
10.0 9.5 9.0 8.5 8.0 7.5 7.0 6.5 6.0 5.5 5.0 4.5 4.0 3.5 3.0 2.5 2.0 1.5 1.0 0.5 0.0
f1 (ppm)

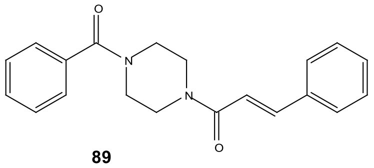

89

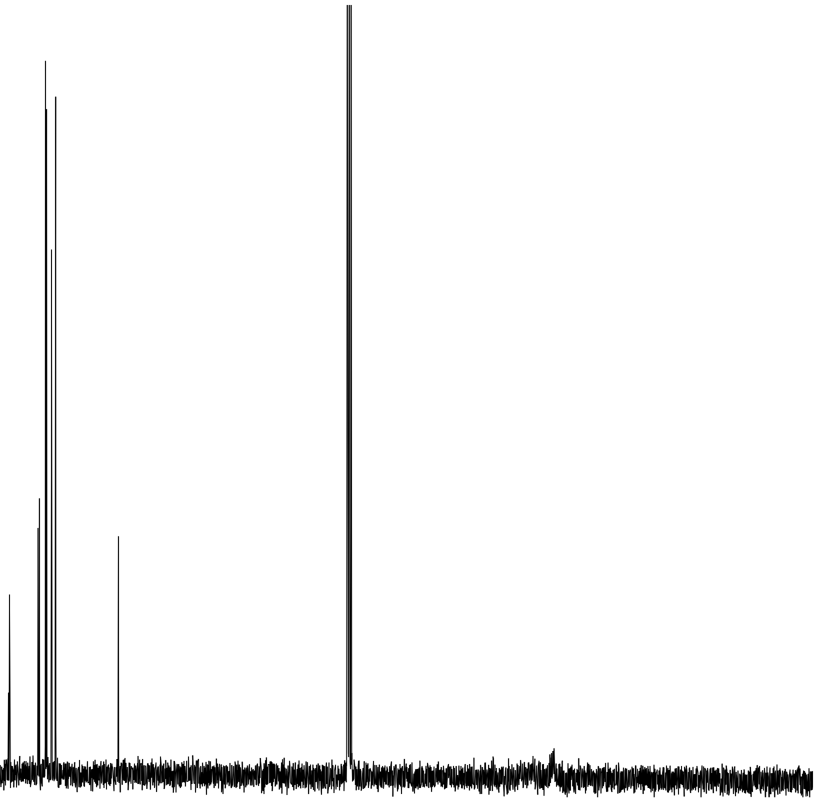

200 190 180 170 160 150 140 130 120 110 100 90 80 70 60 50 40 30 20 10 0
f1 (ppm)

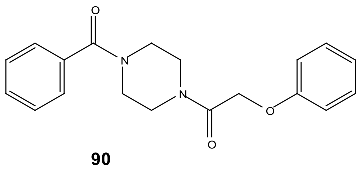
O
N
N
O
O

90

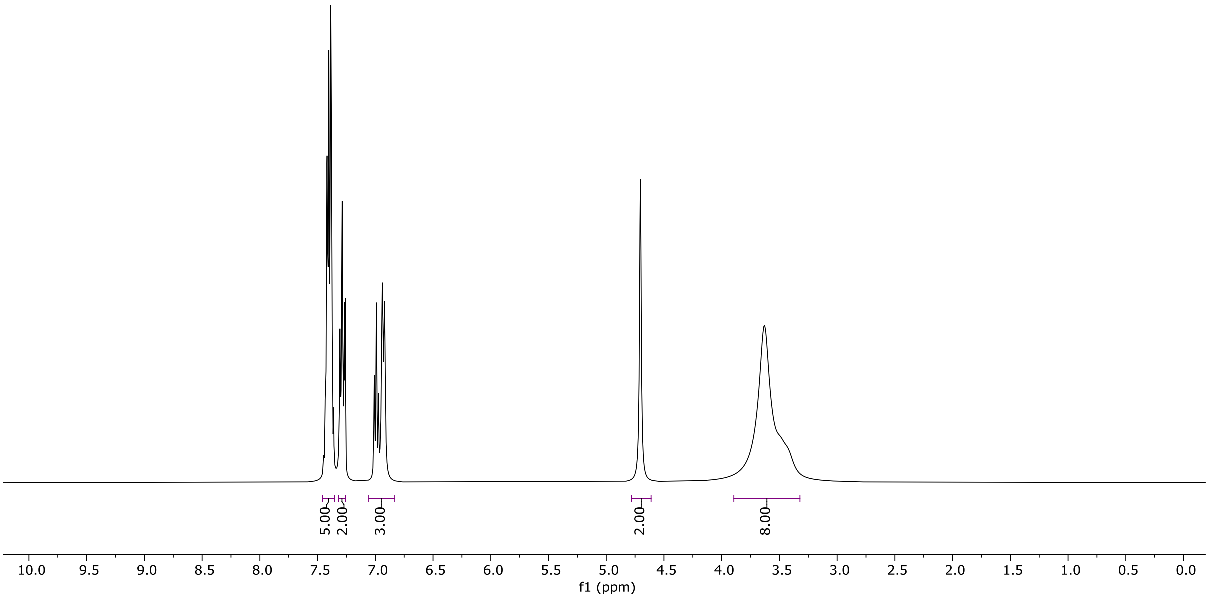
5.00
2.00
3.00
2.00
8.00
10.0 9.5 9.0 8.5 8.0 7.5 7.0 6.5 6.0 5.5 5.0 4.5 4.0 3.5 3.0 2.5 2.0 1.5 1.0 0.5 0.0
f1 (ppm)

90

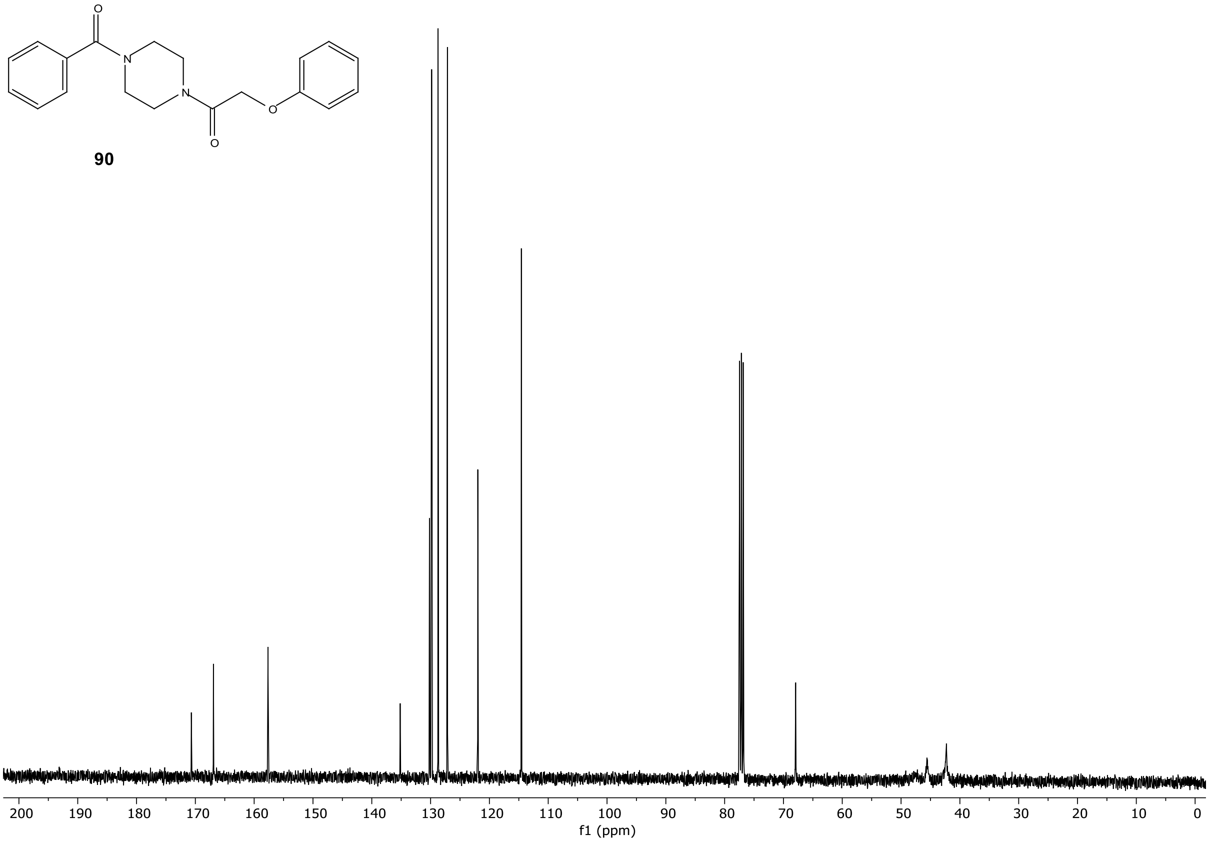
O
N
N
O
O
200 190 180 170 160 150 140 130 120 110 100 90 80 70 60 50 40 30 20 10 0
f1 (ppm)

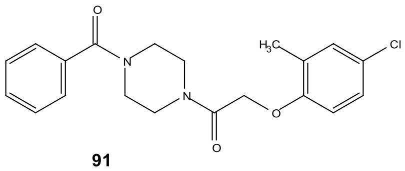

91

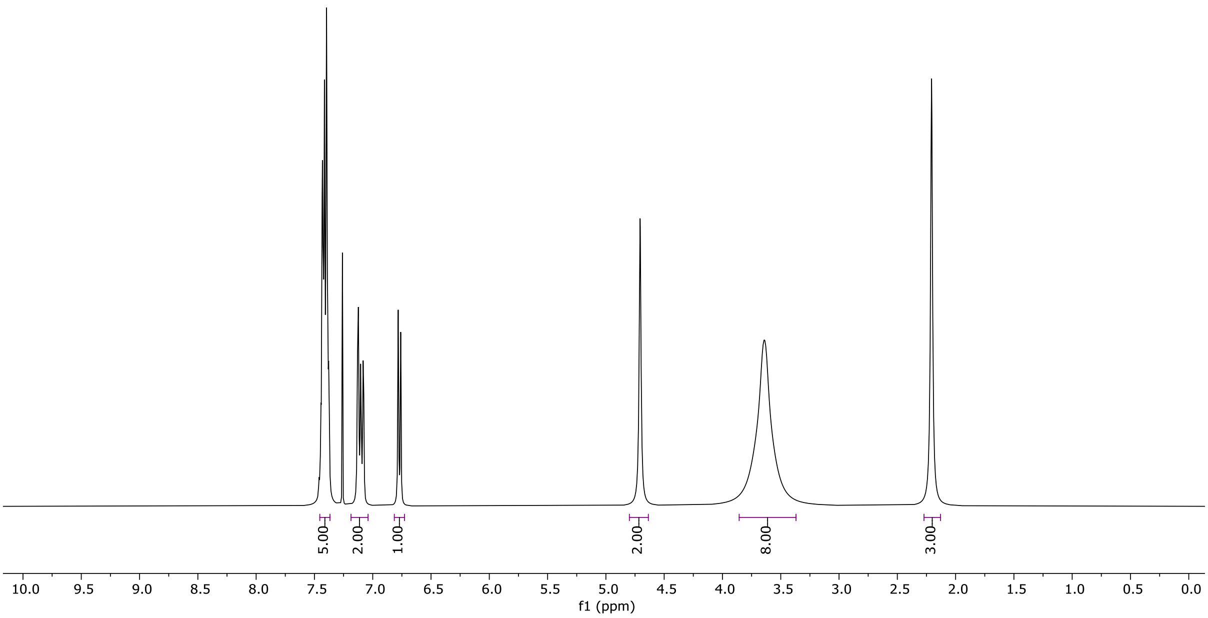
5.00
2.00
1.00
2.00
8.00
3.00
10.0 9.5 9.0 8.5 8.0 7.5 7.0 6.5 6.0 5.5 5.0 4.5 4.0 3.5 3.0 2.5 2.0 1.5 1.0 0.5 0.0
f1 (ppm)

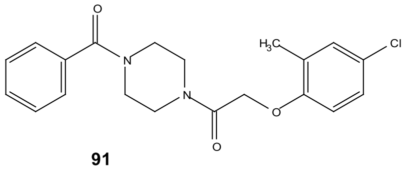

91

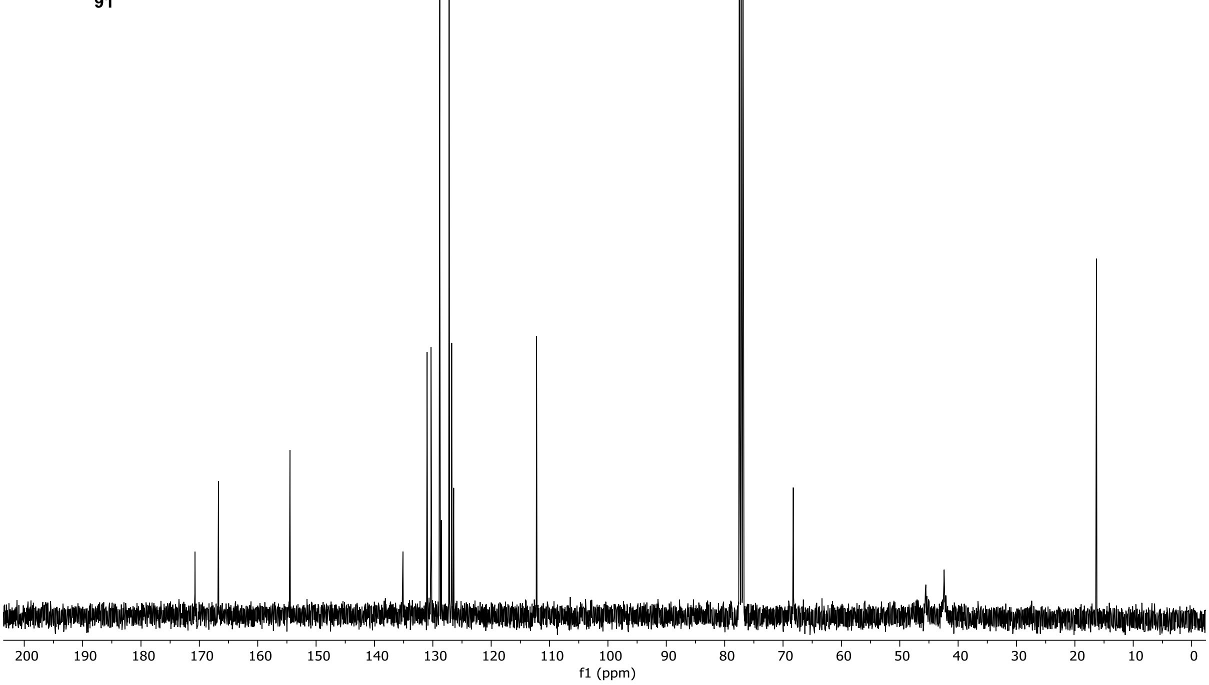
200 190 180 170 160 150 140 130 120 110 100 90 80 70 60 50 40 30 20 10 0
f1 (ppm)

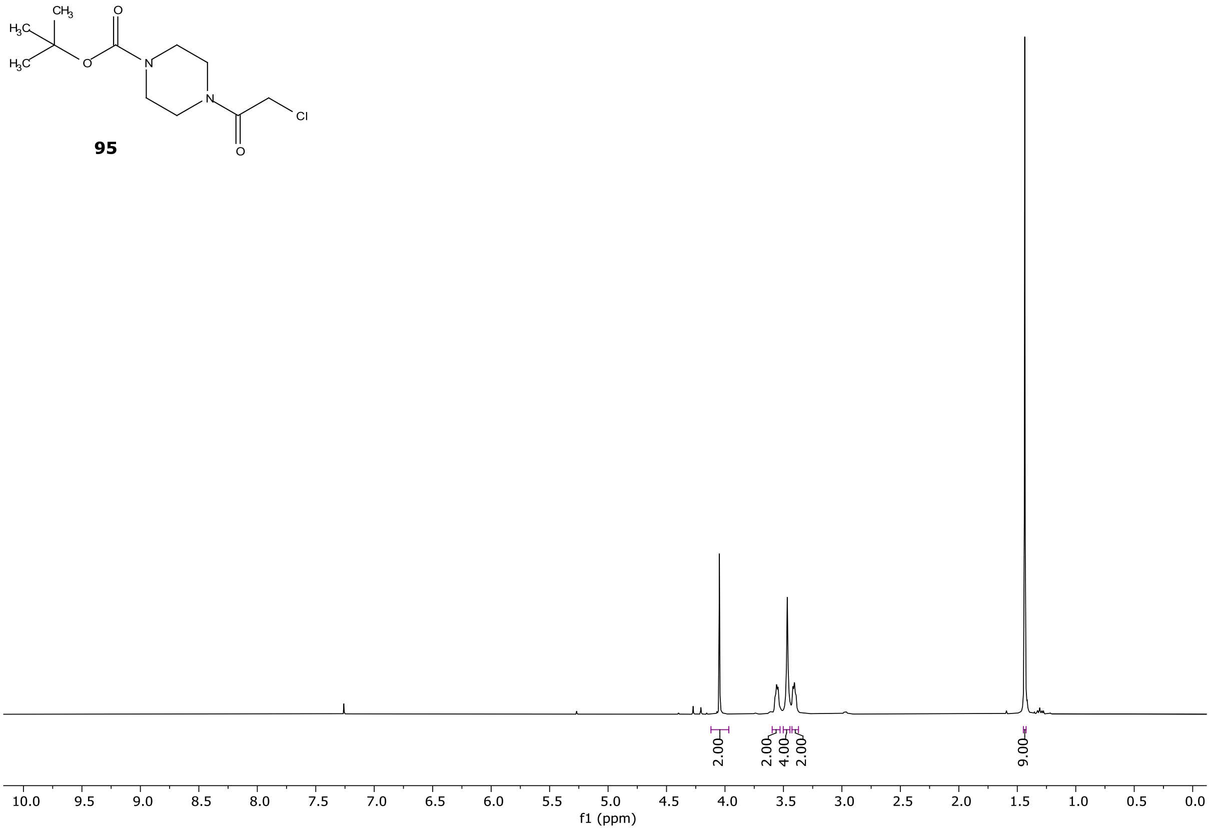

H_3C
CH_3
H_3C
O
O
N
N
O
Cl
2.00
2.00
4.00
2.00
9.00
10.0 9.5 9.0 8.5 8.0 7.5 7.0 6.5 6.0 5.5 5.0 4.5 4.0 3.5 3.0 2.5 2.0 1.5 1.0 0.5 0.0
f1 (ppm)

95

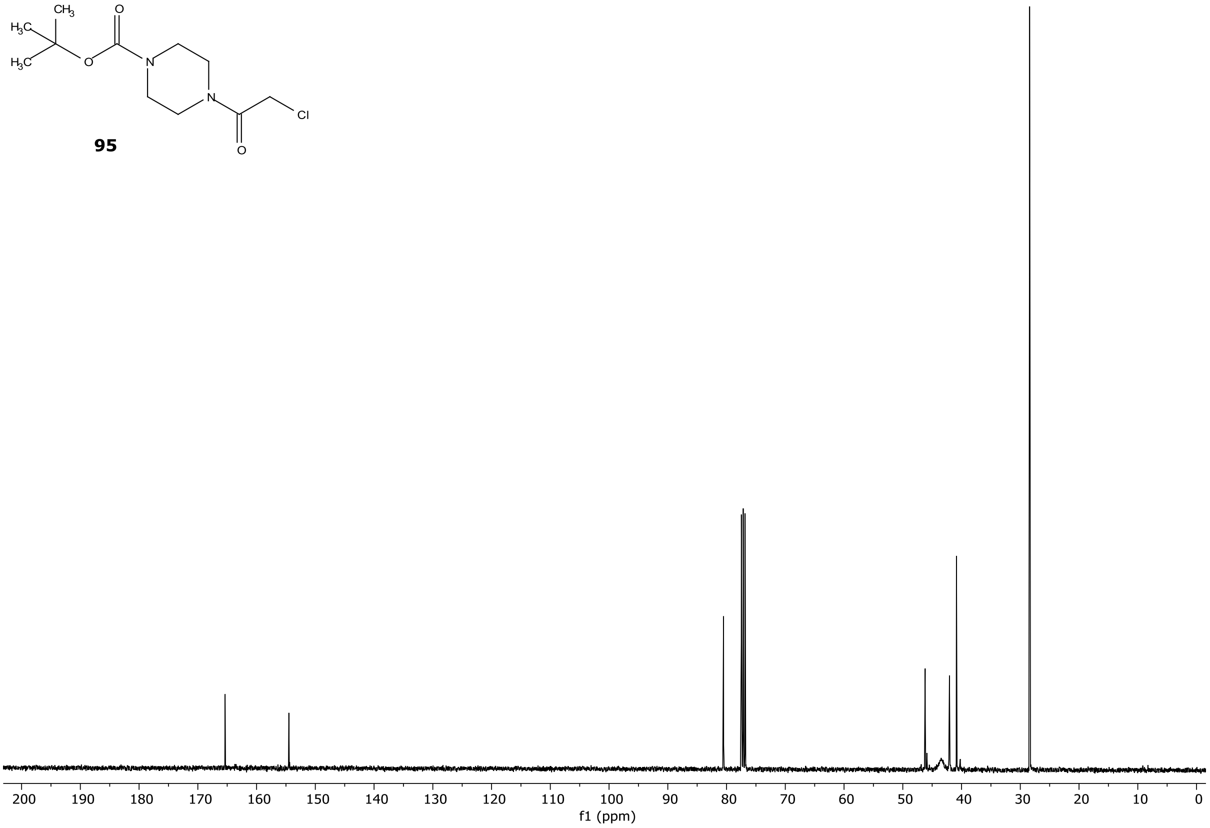

H_3C
CH_3
H_3C
O
O
N
N
O
Cl
200 190 180 170 160 150 140 130 120 110 100 90 80 70 60 50 40 30 20 10 0
f1 (ppm)

95

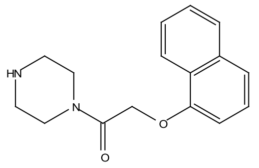

96

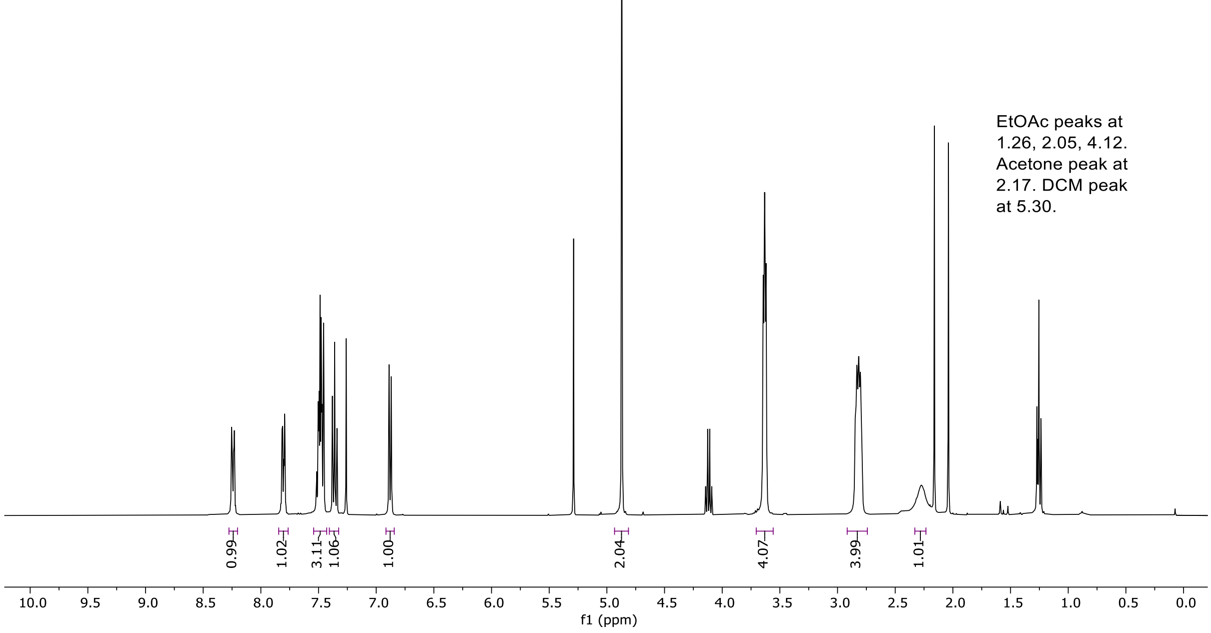
EtOAc peaks at 1.26, 2.05, 4.12. Acetone peak at 2.17. DCM peak at 5.30.
0.99
1.02
3.11
1.06
1.00
2.04
4.07
3.99
1.01
10.0 9.5 9.0 8.5 8.0 7.5 7.0 6.5 6.0 5.5 5.0 4.5 4.0 3.5 3.0 2.5 2.0 1.5 1.0 0.5 0.0
f1 (ppm)

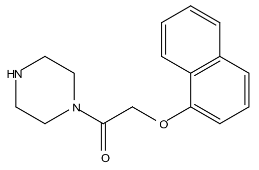

96

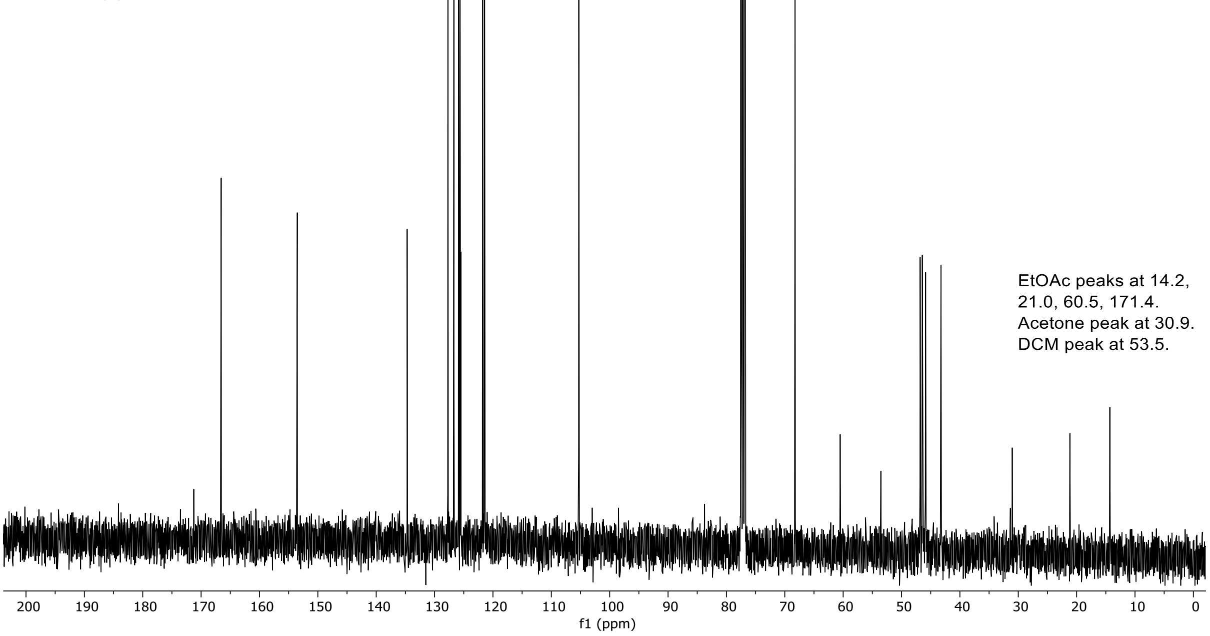
EtOAc peaks at 14.2, 21.0, 60.5, 171.4. Acetone peak at 30.9. DCM peak at 53.5.
200 190 180 170 160 150 140 130 120 110 100 90 80 70 60 50 40 30 20 10 0
f1 (ppm)

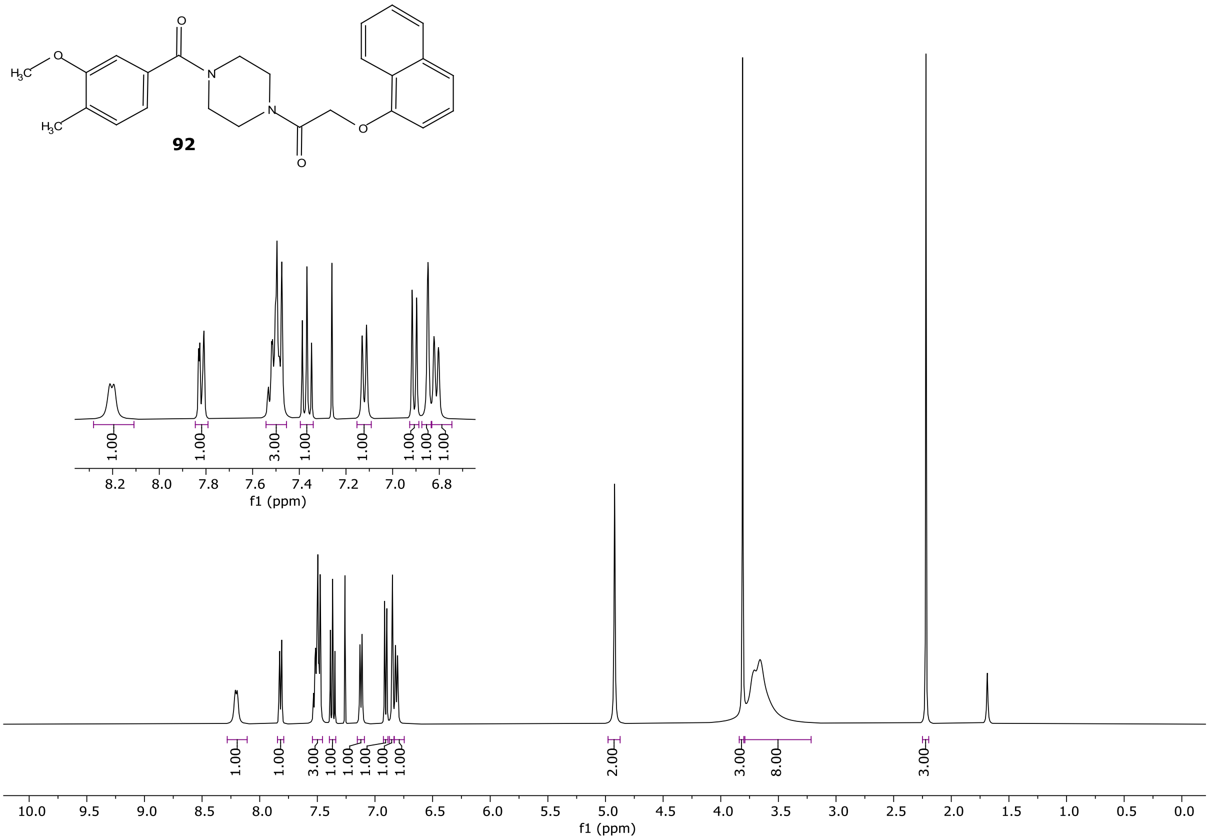

92

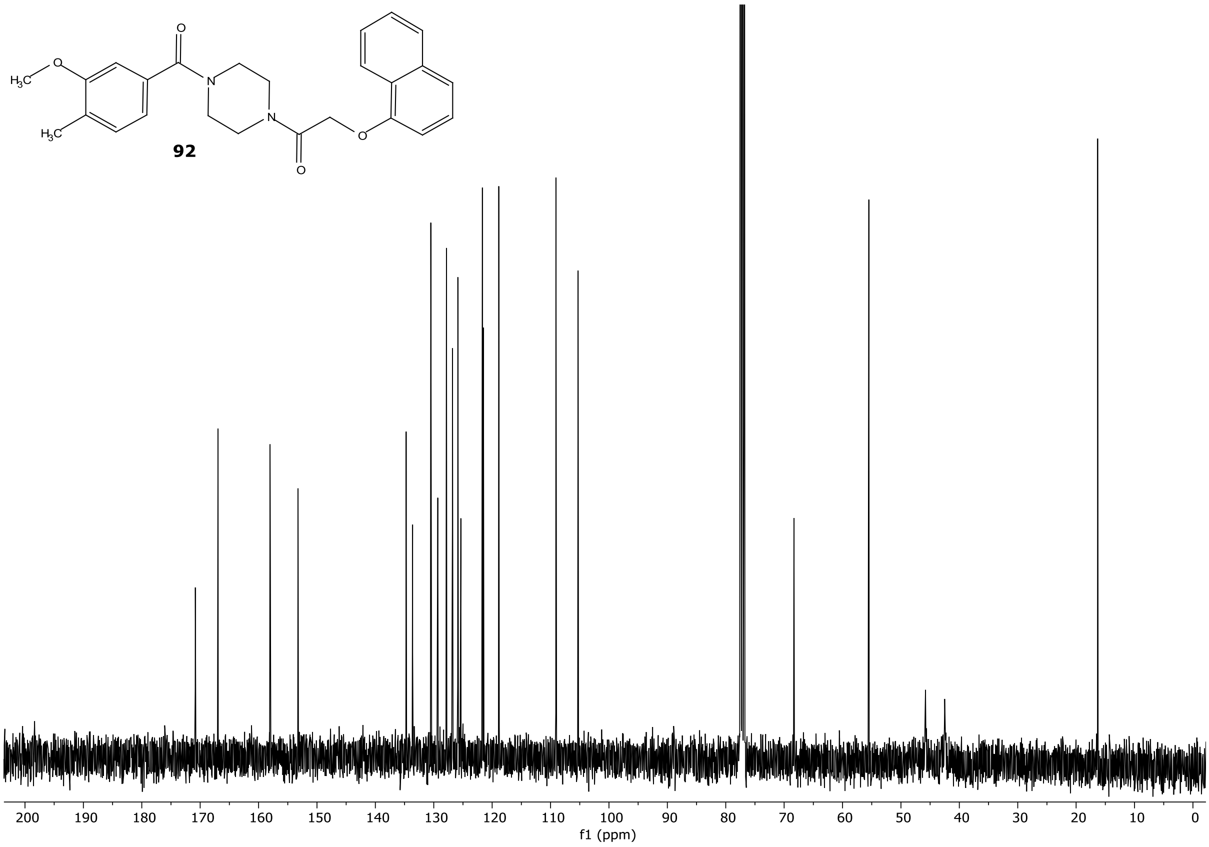

92
